# Soil features in rookeries of Antarctic penguins reveal sea to land biotransport of chemical pollutants

**DOI:** 10.1371/journal.pone.0181901

**Published:** 2017-08-16

**Authors:** Anna C. Santamans, Rafael Boluda, Antonio Picazo, Carlos Gil, Joaquín Ramos-Miras, Pablo Tejedo, Luis R. Pertierra, Javier Benayas, Antonio Camacho

**Affiliations:** 1 Instituto Cavanilles de Biodiversidad y Biología Evolutiva, Universitat de Valencia, Paterna, Spain; 2 Departament de Biologia Vegetal, Facultat de Farmàcia, Universitat de València, Burjassot, València, Spain; 3 Escuela Politécnica Superior, Departamento de Agronomía, Universidad de Almería, Almería, Spain; 4 Departamento de Ecología, Facultad de Ciencias, Universidad Autónoma de Madrid, Madrid, Spain; 5 Departamento de Biogeografía y Cambio Global, Museo Nacional de Ciencias Naturales, Madrid, Spain; Centre National de la Recherche Scientifique, FRANCE

## Abstract

The main soil physical-chemical features, the concentrations of a set of pollutants, and the soil microbiota linked to penguin rookeries have been studied in 10 selected sites located at the South Shetland Islands and the Antarctic Peninsula (Maritime Antarctica). This study aims to test the hypothesis that biotransport by penguins increases the concentration of pollutants, especially heavy metals, in Antarctic soils, and alters its microbiota. Our results show that penguins do transport certain chemical elements and thus cause accumulation in land areas through their excreta. Overall, a higher penguin activity is associated with higher organic carbon content and with higher concentrations of certain pollutants in soils, especially cadmium, cooper and arsenic, as well as zinc and selenium. In contrast, in soils that are less affected by penguins’ faecal depositions, the concentrations of elements of geochemical origin, such as iron and cobalt, increase their relative weighted contribution, whereas the above-mentioned pollutants maintain very low levels. The concentrations of pollutants are far higher in those penguin rookeries that are more exposed to ship traffic. In addition, the soil microbiota of penguin-influenced soils was studied by molecular methods. Heavily penguin-affected soils have a massive presence of enteric bacteria, whose relative dominance can be taken as an indicator of penguin influence. Faecal bacteria are present in addition to typical soil taxa, the former becoming dominant in the microbiota of penguin-affected soils, whereas typical soil bacteria, such as Actinomycetales, co-dominate the microbiota of less affected soils. Results indicate that the continuous supply by penguin faeces, and not the selectivity by increased pollutant concentrations is the main factor shaping the soil bacterial community. Overall, massive penguin influence results in increased concentrations of certain pollutants and in a strong change in taxa dominance in the soil bacterial community.

## Introduction

**Fig 1:**
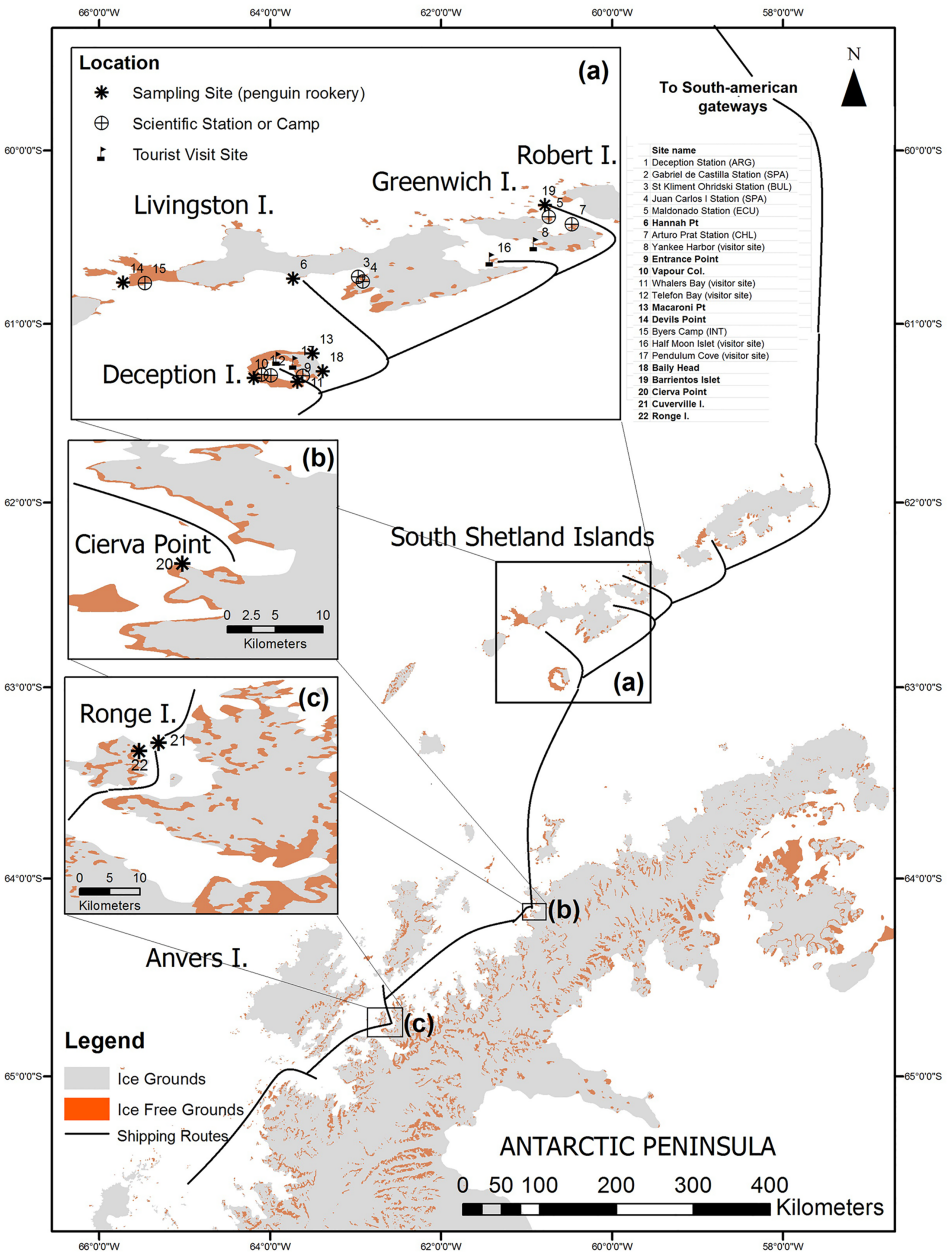
Spatial display of the sampling sites and frequent maritime routes within the South Shetlands Islands [1]. All human activity locations in the region and their condition (i.e. touristic site, scientific station, specially protected site) are also listed, with the sampled penguin rookeries highlighted in bold.

Antarctica presents a relatively high isolation originated by the circumpolar atmospheric and oceanic currents that make it an ideal place to develop studies on dispersion of global pollutants. Its extreme climatic conditions have also greatly limited the development of human activities in this area until recent years [1]. Therefore, it offers a unique opportunity to establish baseline levels for certain contaminants [2], also being an excellent monitoring area to develop referential studies aimed at identifying and recording these levels. However, in recent years, some studies have suggested that Antarctica is no longer a pristine environment due to the gradual emergence of certain pollutants from various sources [3,4,5], which have been measured in different environmental matrices [6,7]. Considering anthropogenic causes, the origin of the increase of the concentration of these chemical substances can be due to long-distance transport, i.e. persistent pollutants transported from other parts of the world [3,8,9], or may result from local activities such as fishing, tourism or research [5,10]. Relatively high levels of metal concentrations have been reported from several sites of the maritime Antarctica, partly resulting from the natural geochemical activity occurring in this region [3,11,12], but they could also be a consequence of cumulative human activity [13]. The natural input may have been amplified by global and local anthropogenic activities such as global industrialization and air pollution via atmospheric and oceanic circulation and deposition, as well as by regional maritime shipping, oil spills, debris, sewage, and fuel combustion, among others [14]. Currently, the maritime Antarctic region concentrates a great amount of human activities compared to the rest of Antarctica, such as the presence of many scientific stations as well as flying and shipping operations [1] mainly related to scientific research and tourism (Fig 1). The input of pollutants from these sources, although appears to be very low in a continental context, could have a significant effect on the bioaccumulation by the local biota, already affecting some endemic species [5]. Therefore, the quantification of natural baseline levels of metals in the Antarctic environment became an important issue for the international scientific community [15]. In line with this growing need, several trace element studies have been undertaken in Antarctica in different environmental compartments (water, soils, sediments, snow and biota), generating a basic knowledge about the background values and the impact of human activities regarding this issue [7,13,15,16,17,18,19,20,21,22].

The possible increase of metal concentration in Antarctic soils via penguin excreta has been suggested by several authors [16,17,23,24,25], though its influence on both the chemical and biological features of the affected soils was not jointly demonstrated so far. Penguins are placed in a high trophic level within the food web, and they are potential sentinels of pollution as they can be easily monitored, have a wide-range, and are abundant and long-living [26]. They usually form large breeding colonies (rookeries) that hold tens of thousands individuals, and they feed almost exclusively in the sea, but nest on land. Furthermore, because birds are able to eliminate metals through excrements [2], penguins can potentially act as biotransporters of chemical elements from marine to terrestrial ecosystems [27], thus concentrating contaminants that are bioaccumulated and biomagnified through the marine food web [28]. Some evidences already indicate that the Antarctic coastline is often highly polluted [29], presenting, in some sites, high concentrations for certain metals that could end up in terrestrial ecosystems through penguin excreta, feathers, eggshells and dead bodies. Moreover, recent studies based on the use of porphyrins profile alterations as a marker of biochemical effects also have demonstrated negative effects on Antarctic penguins eventually associated to metal pollution [30,31]. Thus, the monitoring of the levels of metals in ornithogenic soils transported by penguins is an important task for the assessment of the environmental health in Antarctica.

In this study we sampled different penguin rookeries located in deglaciated areas [32,33] of the maritime Antarctic region in order to analyse the concentration of chemical elements in surface soils and to describe the associated soil microbiota. Sampling was approved under the regulations of the Scientific Committee of Antarctic Research (SCAR) through the Spanish Polar Committee mediation. The hypothesis tested here was that it would be expected having significant differences in the pollutant concentrations and soil microbiota within rookeries (ornithogenic soils) compared to the typical levels and microbiota present in nearby ‘control’ areas (non-ornithogenic soils), as a direct consequence of the biotransport by penguins from sea (ingestion) to land (defecation). Some basic physical and chemical properties of the sampled soils were determined to assist with the consideration of co-factors affecting the interpretation of the results. Potential differences in element biotransport trends due to geographical (South Shetland Islands vs Antarctic Peninsula) and biological effects (two penguin species, Gentoo penguin vs Chinstrap penguin) were also tested. In addition, the composition and diversity of soil microbiota was also studied for some locations by molecular techniques. The purpose of these additional analyses was to assess if the eutrophication and the enrichment in certain pollutants related to the presence of penguin colonies could also drive changes in the soil microbial community, and whether this was affected by biological contamination from penguin excreta. This examination would serve to address whether the composition of soil microbiota could be used as an additional indicator of the sources of pollution. Altogether, the exploration of the effects on all the studied parameters can contribute to determine the type and impact of anthropogenic pollution on Antarctic biota and ecosystems, such as those impacted by human transports [34,35], thus further improving the knowledge on the biogeochemical processes occurring in these supposedly pristine regions.

## Material and methods

### Sampling sites and experimental design

Field samples were taken during the 2012 Antarctic austral summer (from late January to mid February). In order to visualize the spatial context of the sampling sites Fig 1 was created from baseline spatial cartography available from the Scientific Committee on Antarctic Research (SCAR) Antarctic Digital Database (ADD) rock outcrop layer (Version 7 – www.add.scar.org). Up to 46 soil samples were taken from the upper 10–15 cm layer at 10 different locations (Table 1 and Fig 1), distributed all within the maritime Antarctic region, covering penguin breeding colonies (rookeries) of the genus *Pygoscelis*, either of Gentoo (*P*. *papua* Forster, 1781) or Chinstrap (*P*. *antarctica* Forster, 1781) penguins.

**Table 1:** List of samples collected in each site and sample design applied.

Area	Site	Coords.	Species	No of Samples	Ornithogenic Soils	Non ornithogenic soils	Sample Design
South Shetland Islands	Byers Peninsula (Devil´s Point). (BY)	62°39’55”S61°09’40”W	Gentoo	6	BY1, BY3, BY5	BY2, BY4, BY6	Linear sampling
	Hannah Point (PH)	62°39’14”S60’36’44”W	Gentoo	2	PH2	PH4	Linear sampling
	Hannah Point (PH)	62°39’11”S60’36’23”W	Chinstrap	2	PH1	PH3	Point sampling
	Barrientos Island (BR)	62°24’22’S59°44’27”W	Chinstrap	3	BR2, BR3	BR1	Linear sampling
	Barrientos Island (BR)	62°24’25”S59°44’27”W	Gentoo	2	BR5	BR4	Linear sampling
Deception Island (South Shetland Islands)	Vapour Col. (CV)	62°59’28”S60°43’22”W	Chinstrap	12	CV2, CV3, CV4, CV7	CV1, CV5, CV6, CV8 to CV12	Concentric
	Baily Head (MB)	62°57’46”S60°30’09”W	Chinstrap	6	MB3, MB5	MB1, MB2, MB4, MB6	Linear sampling
	Macaroni Pt (PM)	62°53’58”S60°31’57”W	Chinstrap	3	PM2	PM1, PM3	Linear sampling
	Entrance Pt (PE)	62°59’57”S60°33’51”W	Chinstrap	4	PE2, PE4	PE1, PE3	Linear sampling
Antarctic Peninsula	Cierva Cove (CC)	64°09’45”S60°54’05’S	Gentoo	2	CC1, CC2	-	Linear sampling
	Cuverville Island (CU)	64°41’30”S62’37’28”W	Gentoo	2	CU1, CU2	-	Linear sampling
	Ronge Island (RO)	64°41’51”S62°38’46”W	Gentoo	2	RO1, RO2	-	Linear sampling

These penguin species are characterized by their wide distribution, not exclusively Antarctic, being also present in Sub-Antarctic areas and, in the case of Gentoo penguin, even in Patagonia. Sampling locations can be subdivided in two main areas: a) the South Shetland Islands archipelago, with 7 sites (4 in Deception Island), and b) the northern Antarctic Peninsula, with 3 sites (Fig 1). There was a general lack of clear soil horizon differentiation, which made impossible to establish different layers. A short description of the studied sites is given in S1 Table.

Three complementary sampling strategies were used, being the main one a 'lineal' survey carried out from the coastline to the nearest hillside while crossing the penguin colony. Samples taken directly immersed in the colony are referred as 'ornithogenic soils' and as such indicated in Table 1. This approach was carried out in 9 sites. Alternatively, one site, Vapour Col (CV), had a more extensive 'concentric' sampling design, with 12 samples collected at three increasing distances from the centre of the colony in all four directions. In addition, due to its small extent there is a 'point' sample taken in a small rookery of chinstrap immersed into a Gentoo colony in Hannah Point (PH). Contrarily to the sampling locations at the South Shetland Islands, which included both penguin affected and unaffected soils, there was virtually impossible taking samples of ‘non-ornithogenic soils’ (soils lowly or not affected by penguins) for sites located in the Antarctic Peninsula because all the ice-free areas were occupied to a greater or lesser extent by penguin colonies. The soil sample collection was performed using sterile instruments. Samples were put in sterile plastic bags, and then stored frozen until the moment of the sample processing for analyses.

### Soil physical-chemical analysis

The pH was measured in a 1:5 fresh soil-distilled water extract, after shaking for 5 minutes and then left for two hours [36], thereafter pH was measured with a micropH 2002 Crison pH-meter. The rest of physical-chemical analyses were performed on the dried samples. For these, subsamples were dried during c.a.48 h at 40°C, crushed, and sieved through a 2 mm mesh. Then a fraction of this treated sample (c.a. 5 g) was pulverized with an agate mortar and mixed with a Frist shaker (Centrifugal Ball Mill, mortar Pulverisette-6, Fritsch), then sieved again through a 0.5 mm mesh prior to element analysis determination. Electrical conductivity (EC) was measured in an aqueous soil extract at a 1:5 ratio (w/v) [37] using a Crison conductivity meter. To determine the presence of CaCO_3_ a pre-test consisting in adding two drops of HCl 1:1 (v/v) to 1 gram of soil was performed. Since no evident reaction was observed in any of the samples, the analysis of CaCO_3_ was not further performed. Total C and N concentrations were analyzed on a LECO TRU-SPEC CN analyzer (Leco Corp.), then these results were used to determine the % in weight of C and N in soil. The % weight of organic carbon (%Corg) was determined using the same method but on previously acidified samples to remove inorganic carbon. Total P was determined by ICP on digested samples as described below for the elementary analysis. C, %Corg, and N analyses, as well as pH and EC measurements, were replicated (S3 Table).

The elements concentrations were determined using an inductively coupled plasma optical emission spectrometer (ICP-OES, THERMO ICAP 6500 DUO, Waltham, MA, USA). To perform the analyses 0.1 grams of the dry pulverised sample were put in the digester and 4 mL de HNO_3_ PA-ISO 69% and 1 mL de H_2_O_2_ 33% (v/v) were added. The tube was brought to the reactor with a ramp of increasing temperature and kept for 20 minutes at 220°C. Once the tubes were cold after digestion, milliQ water was added to a final volume of 25 mL and the extracts were measured by ICP. This method can be considered a pseudo total analysis accounting for the “environmentally available elements” [38], as it is not a total digestion technique for most samples. Instead, it is a very strong acid digestion that dissolves almost all elements that could become “environmentally available”. By design, elements bound in silicate structures are not normally dissolved by this procedure as they are not usually mobile in the environment. The elements analysed were Na, K, Ca, Mg, Fe, Cu, Mn, Zn, B, P, S, Al, Pb, Cd, Cr, Ni, As, Be, Bi, Co, Li, Mo, Sb, Se, Sr, Ti, Tl, and V, from which the concentrations of the most significant elements for our study are giving. The element concentrations were analysed as single samples on a dry-mass basis.

The quality assurance and control procedures were implemented through the analysis of a standard reference material CMR044-050 Lot n° CF044 (chalky loam soil), obtained from National Institute of Standards and Technology (Laramy, WY, USA). Reagent blanks were monitored throughout the analysis and were used to correct the analytical results. The recovery rates (%) obtained for the studied elements were: Al 108±6, As 79±3, Cd 85±4, Co 91±6, Cu 92±2, Fe 99±8, Mn 107±2, Mo 68±2, Pb 93±9, Se 64±2 and Zn 94±9. A comparison of our results with those given by USEPA [38] and other works [39] is shown in the S2 Table. The relatively lower recovery values obtained for As, Mo and Se may be due to the analytical method used, as ICP-OES is not the most sensitive analytical method for these elements. The quantification limits obtained (mg kg^-1^) are as follows: 2 for Al and Fe; 0.50 for Co, and Se, and 0.10 for As, Cd, Cu, Mn, Mo, Pb and Zn. The uncertainty (%) of the method for each element is as follows: Al 5.4, As 5.6, Cd 4.6, Co 6.0, Cu 4.1, Fe 3.9, Mn 6.2, Mo 6.1, Pb 6.2, Se 6.5 and Zn 5.0.

### Molecular analyses

In recent years, techniques based on DNA extraction from soils and its selective amplification by PCR have been widely used to study the microbial communities of edaphic systems and their biodiversity [40,41]. When multiple samples have to be compared, as in our case, soil diversity have often be assessed by means of denaturing gradient gel electrophoresis (DGGE) and community fingerprinting, which helps to discriminate the composition of the microbial communities in each sample, then complemented by Sanger sequencing of the main bands. In our case the bacterial diversity patterns can be compared to determine the influence of soil features and pollution in the community composition of the studied soils. In order to avoid any possible bias due to the distance and geological features among the sampling sites, the study of the soil microbiota was centred on samples from Deception Island, which present similar soils and thus the differential effect of penguins on soil microbiota can be tested.

For DNA extraction and purification, 1 gram of each fresh melted sample was processed using a commercially available kit (E.Z.N.A.^™^ Soil DNA Kits, Omega Bio-Tek) following the manufacturer’s protocol. After the extraction, a PCR amplification of the 16S rDNA fragments for bacteria was made. The mix per reaction consisted in: 2 μl of 10X Tris HCl buffer, 0.8 μl of MgCl_2_ 50 mM, 0.4 μl of dNTPs 10 mM, 2 μl of each primer, 1 μl of BSA, 0.5 μl of polymerase, 10.3 μl of Milli-Q ultrapure water and 1 μl of the DNA sample. The primers used were 341F-GC (5’-CGC CCG CCG CGC GCG GCG GGC GGG GCG GGG GCA CGG GGG GCC TAC GGG AGG CAG CAG-3’) and 534R (5´-ATT ACC GCG GCT GCT GG-3´). The program setting was 5 min at 94°C, 1 min at 80°C, 35 cycles at 94°C 1 min, 45°C 1 min and 72°C 1 min, and a final elongation step at 72°C for 30 min [42]. All the amplifications were carried out in an Eppendorf Mastercycler Personal thermocycler. Amplification of the PCR product was checked by agarose gel electrophoresis (2% agarose, 120 V, 40min) by taking 2 μl of the PCR product, 2 μl of the loading buffer and 5 μl of SYBR-Green and placing a marker in the gel to verify the size of the amplified bands.

Once amplification was confirmed, a DGGE analysis was performed for each of the different sample sites using a CBS DGGE System (CBS Scientific Company). 18 μl of the PCR product were loaded on a 7% polyacrylamide gel (Acrylamide/Bisacrylamide 37.5:1) containing a denaturant gradient of 40–70% made by urea and formamide. Gels were electrophoresed at 60°C at a constant voltage (250 V) for 5 h and were stained for 40 minutes using SYBR-Green. Bands were recorded to digital images by UV light gel transillumination.

Nucleotide sequences of DNA fragments recovered from bands on DGGE gels were determined by excising the bands from a DGGE gel with an adapted 1-ml pipet tip and the DNA was then eluted in 25 μl sterile water at 4°C overnight. The DNA fragment was amplified from the eluted solution by PCR and the mobility on DGGE gels was checked. The primer pair without GC clamp (341F and 534R) was used in the template amplification by PCR. DNA was sent for Sanger sequencing to Macrogen Sequencing Service (Macrogen Inc., Korea). Possible chimeric sequences were screened using Ribosomal Database Project (RDP release 8.1) online Chimera Check program (http://rdp8.cme.msu.edu/html/analyses.html) [43]. Taxonomic identity of each phylotype was determined using a naive Bayesian rRNA classifier described in the Ribosomal Database Project RDP Classifier 2.0, a with a 50% bootstrap threshold [44].

### Data analyses

All data are directly supplied in the manuscript or as supplementary material. For the statistical analyses, the concentrations of elements below the detection limit were substituted by values one-half of the detection limit. After a preliminary correlation analysis, multivariate analyses were centred on data of significant elements, and those elements uncorrelated (either positively or negatively) with the percentage of organic carbon (%Corg), the main descriptor of penguin activity, were not used for the multivariate analysis. This resulted in the removal of Na, K, Ca, Mg, B, S, Cr, Ni, Be, Bi, Li, Sb, Sr, Ti, Tl and V from further multivariate analyses. A principal component analysis (PCA) was then carried out using log-transformed data of selected physical (pH, EC) and chemical (%C, %Corg, %N, P, and concentrations of Al, As, Cd, Co, Cu, Fe, Mn, Mo, Pb, Se and Zn) features of the soil samples. Since variances were non-homogeneous, non-parametric tests (Median test and Kruskal–Wallis one-way analysis of variance) were applied to identify differences in soil composition between ornithogenic and non-ornithogenic soils, rookeries of Gentoo and Chinstrap penguins, and sites located either in the South Shetland Islands or in the Antarctic Peninsula, respectively. Significance level for null hypothesis rejection was established in 0.05 for all tests. Bivariate correlations were also performed for selected metals concentrations against the % organic C, the latter as an indicator of organic matter content and thus of penguin influence. The software used for the statistical analysis was SPSS Statistics 21 (SPSS, Inc.).

Data obtained from rookeries were further compared with those of non-ornithogenic soils (control areas) through two approaches. The first was the comparison of the arithmetic mean, the standard deviation, and the 95% confidence interval about the mean of the metal concentration, with the estimated background range (estimated as the mean plus or minus two standard deviations). The second was the use of the *biogenic enrichment factor* (BEF), a metric used to rank the elements based on the likelihood that they are enriched by penguin presence and activity. Following Brimble et al. [45], this indicator was calculated as the ratio of the average level of each parameter within the rookeries divided by the average level of the same parameter within the control areas.

Finally, using the results of the DGGE profiles for Bacteria, a dissimilarity matrix based on the Jaccard coefficient was calculated and a dendrogram was built using the matrix with the presence or absence of bands [46,47,48]. A dendrogram for each site was drawn using the Bio-Rad *Quantity One* software.

## Results

### Field site observations

All sampling sites immersed within the rookeries were characterized for the dominance of the algae *Prasiola crispa* Meneghini, growing in rock surfaces or particularly in compressed soils. Nearby ‘control’ locations not directly affected by ornithogenic soils (mainly uphill) comprised several cryptogamic species typical of the region (i.e. *Sanionia uncinata*, *Polytrischastrum alpinum*) and the two Antarctic vascular plants (*Deschampsia antarctica* and *Colobanthus quitensis*). All rookeries were relatively flat and coastal (0–20 m altitude) with the exception of those from Deception Island (Chinstrap penguin sites), which had steeper slopes and altitude around 20–40 m. for Vapour Col and Baily Head and 40–60 m for Macaroni Point and Entrance Point. In addition, the pyroclastic substratum of this volcanic island was different to the rest (more coarse).

### Element concentrations and organic matter content: Differences between ornithogenic and non-ornithogenic soils

The main soil features of the sampled sites were first explored. All samples correspond to Cryosols according to the World Reference Base of soils Resources, specifically to Ornithogenic Gelisols in the case of rookeries. Table 2 shows the most important physical and chemical features of the studied soils, paying special attention to the percentage of organic carbon (%Corg), since its abundance is directly related to the intensity of the influence of penguins. As for other related parameters, such as the percent in weight of C and N and P, the %Corg also showed much higher values in samples having more ornithogenic influence, with organic C accounting for most of the soil carbon content in these samples. In Vapour Col (CV), soils from the two sites located closer to the centre of the colony (CV2 and CV3 samples) had by far the highest organic carbon concentrations, reaching values in CV3 as high as more than 10% of total weight made by C and more than 5% of N. Meanwhile, the rest of the samples decreased its organic content accordingly to their peripheral location within the penguin colony. A similar pattern was found among the samples from other sites in Deception Island (Baily Head, Macaroni Point and Entrance Point). Among these, the ornithogenic samples (PE-2 and PE-4) of Entrance Point showed higher values (up to 7.2% C and 3.5% N) than those of the other two sites (maximum values lower than 2.9% C and 0.8% N). Similar results, with much higher values for the ornithogenic soils, were obtained for these variables for the rest of studied sites both in the South Shetland Islands and, especially, in the Antarctic Peninsula, the later always corresponding to ornithogenic soils. Concerning electrical conductivity, soil samples with higher ornithogenic impact generally presented higher values than non-ornithogenic samples from the same location. Similarly, non-ornithogenic soils generally showed less acidic pH than ornithogenic soils. All samples are characterized by a narrow C:N ratio, which implies low decomposition rates, a scarce fertility, and the predominance of the mineralization processes for the soil organic material, presenting nitrogen in excess to that required by the microbial populations

**Table 2:** Basic physical and chemical properties and organic content of the studied soils.

Site	pH	EC	%Corg	%C	%N	%P	C:N ratio
**BY1**	7.16±0.14	2.51±0.07	5.98±0.01	6.37±0.14	2.52±0.04	2.060	2.53
BY2	5.10±0.16	0.20±0.01	3.15±0.07	3.15±0.10	0.52±0.02	0.194	6.06
**BY3**	5.36±0.05	0.40±0.01	6.48±0.03	7.35±0.21	1.32±0.02	0.527	5.57
BY4	5.13±0.13	0.26±0.01	0.82±0.01	0.82±0.03	0.29±0.02	0.151	2.83
**BY5**	5.89±0.15	0.02±0.01	19.05±0.35	20.04±0.57	8.42±0.09	3.775	2.38
BY6	6.18±0.13	1.15±0.07	4.00±0.07	4.42±0.14	1.19±0.02	2.293	3.71
**PH1**	5.48±0.18	3.48±0.11	20.40±0.14	20.91±0.18	10.01±0.19	4.907	2.09
**PH2**	6.21±0.12	0.97±0.02	18.35±0.07	19.03±0.54	10.40±0.04	3.259	1.83
PH3	7.41±0.18	0.27±0.01	0.69±0.01	0.74±0.03	0.27±0.03	0.210	2.74
PH4	9.33±0.14	0.16±0.01	0.13±0.01	0.13±0.01	0.10±0.01	0.050	1.30
BR1	6.74±0.16	0.15±0.01	1.26±0.04	1.27±0.04	0.26±0.03	0.427	4.88
**BR2**	5.02±0.08	0.33±0.02	2.94±0.06	3.30±0.14	0.54±0.05	1.668	6.11
**BR3**	6.66±0.17	4.27±0.42	7.41±0.03	7.42±0.14	3.38±0.05	3.662	2.20
BR4	6.81±0.13	0.18±0.01	1.08±0.04	1.13±0.04	0.30±0.01	0.358	3.77
**BR5**	6.28±0.10	3.46±0.33	7.99±0.27	8.19±0.37	3.10±0.15	4.534	2.64
CV1	7.56±0.16	0.93±0.01	0.70±0.01	0.73±0.03	0.32±0.03	0.303	2.28
**CV2**	7.08±0.14	3.56±0.08	5.74±0.04	5.98±0.14	2.53±0.04	1.141	2.36
**CV3**	6.34±0.14	6.27±0.09	10.45±0.36	10.45±0.35	5.22±0.09	1.899	2.00
**CV4**	7.22±0.14	3.38±0.04	1.51±0.03	1.54±0.07	0.64±0.02	0.602	2.41
CV5	7.19±0.18	0.22±0.01	0.23±0.01	0.23±0.01	0.11±0.02	0.167	2.09
CV6	6.09±0.06	0.28±0.01	0.91±0.02	0.91±0.03	0.23±0.02	0.396	3.96
**CV7**	6.12±0.16	0.77±0.06	2.16±0.05	2.21±0.07	0.52±0.02	0.779	4.25
CV8	6.38±0.11	0.22±0.01	0.55±0.01	0.55±0.01	0.19±0.01	0.215	2.89
CV9	6.27±0.27	0.19±0.01	0.43±0.01	0.45±0.01	0.11±0.01	0.323	4.09
CV10	6.48±0.21	0.08±0.01	0.57±0.01	0.57±0.01	0.16±0.01	0.120	3.56
CV11	6.26±0.28	0.12±0.00	0.34±0.01	0.35±0.01	0.12±0.01	0.275	2.92
CV12	6.00±’.15	0.20±0.01	0.45±0.01	0.47±0.01	0.18±0.02	0.271	2.61
MB1	7.43±0.10	0.82±0.08	0.67±0.01	0.75±0.03	0.27±0.02	0.309	2.78
MB2	7.30±0.12	0.57±0.05	0.66±0.01	0.66±0.01	0.23±0.02	0.245	2.87
**MB3**	6.82±0.10	2.83±0.16	2.43±0.04	2.87±0.14	0.78±0.04	0.436	3.68
MB4	5.45±0.15	0.25±0.01	0.96±0.01	0.98±0.01	0.25±0.01	0.382	3.92
**MB5**	6.81±0.15	2.95±0.15	2.67±0.10	2.78±0.14	0.80±0.04	0.734	3.48
MB6	6.75±0.12	0.14±0.00	0.21±0.01	0.23±0.01	0.10±0.01	0.199	2.30
PM1	7.53±0.16	0.41±0.01	0.65±0.01	0.65±0.01	0.21±0.02	0.098	3.10
**PM2**	6.61±0.13	2.24±0.05	1.74±0.06	1.8±0.01	0.91±0.02	0.372	1.98
PM3	7.91±0.17	0.29±0.01	0.28±0.01	0.28±0.01	0.13±0.02	0.072	2.15
PE1	5.59±0.01	0.27±0.01	0.51±0.01	0.51±0.02	0.21±0.01	0.593	2.43
**PE2**	6.16±0.09	8.34±0.71	7.21±0.01	7.21±0.14	3.51±0.04	1.696	2.05
PE3	8.09±0.13	0.21±0.02	0.32±0.01	0.39±0.01	0.07±0.02	0.073	5.57
**PE4**	6.07±0.07	8.30±0.11	6.93±0.04	7.09±0.13	3.05±0.05	3.517	2.32
**CC1**	5.13±0.15	0.46±0.04	23.90±0.14	24.12±0.11	3.21±0.03	1.863	7.51
**CC2**	6.64±0.07	1.43±0.07	23.40±0.14	24.02±0.02	4.98±0.09	7.616	4.82
**CU1**	6.64±0.01	3.11±0.14	10.75±0.35	10.96±0.49	3.79±0.01	12.781	2.89
**CU2**	6.34±0.18	0.11±0.01	8.25±0.07	8.80±0.14	1.32±0.02	1.779	6.67
**RO1**	5.55±0.14	0.35±0.01	12.30±0.14	12.30±0.28	1.31±0.02	3.312	9.39
**RO2**	6.55±0.15	3.85±0.14	9.50±0.07	9.50±0.01	3.76±0.06	9.921	2.53

Data shown are mean ± standard deviation of replicated samples (except for P, where no replicates were analysed). EC: Electrical Conductivity, in mS/cm; %Corg: % of organic carbon. In bold, samples from ornithogenic soils. C:N ratio calculated from mean values.

The concentrations of the main chemical elements determined by ICP-OES, which showed a higher link, either positively or negatively, with the %Corg in soils, are shown in Table 3. Although the best comparison can be made for soils of the same location thus avoiding the effects of local geology, in general, samples showing higher organic contents (Table 2) due to penguin activity showed much higher levels of certain elements, specifically Cd, Cu, Se, Zn and, in most samples, also As. On the contrary, metals having a marked geochemical origin, such as Al, Co, Fe, Mn and Mo, generally presented a lower relative contribution as organic matter content increased. On the other hand, although the highest lead concentrations were measured in ornithogenic soils of sites located in the Antarctic Peninsula (CC, CU, and RO), when the rest of samples were considered lead did not clearly show higher concentrations in ornithogenic soils of other locations compared to less penguin affected soils (Table 3).

**Table 3:** Element concentrations (mg·Kg^-1^) in the soil of the different sampling sites. In bold, samples from ornithogenic soils.

Site	Al	As	Cd	Co	Cu	Fe	Mn	Mo	Pb	Se	Zn
**BY1**	32232	<0.10	0.29	5.99	40,61	32299	172.30	0.20	5.62	3.09	102.91
BY2	50623	<0.10	<0.10	53,32	72,95	93661	704.79	0.46	5.56	<0.50	82.90
**BY3**	33388	<0.10	<0.10	14,17	52,73	42021	420.60	0.27	5.69	1.73	60.87
BY4	65784	<0.10	<0.10	29,41	40,26	77710	742.12	0.44	5.71	4.15	53.28
**BY5**	47370	3.06	0.71	1,34	94,76	3845	76.78	<0.10	1.31	7.70	120.98
BY6	22333	<0.10	0.57	4,99	81,40	18041	135.90	<0.10	4.19	5.21	107.80
**PH1**	1715	6.49	1.84	0,78	141,99	2522	72.18	<0.10	0.56	11,00	146.25
**PH2**	4295	1.91	0.86	1,99	78,30	5844	109.84	<0.10	1.53	4.56	97.44
PH3	29385	<0.10	<0.10	9,20	21,31	38286	307.26	0.21	4.44	<0.50	57.30
PH4	23500	<0.10	<0.10	7,87	10,43	37688	301.30	0.20	3.55	<0.50	50.23
BR1	38720	<0.10	<0.10	12,90	64,16	29489	193.07	0.39	6.11	<0.50	39.29
**BR2**	36955	<0.10	<0.10	9,62	50,28	28277	193.51	0.35	5.73	6.42	31.39
**BR3**	24881	<0.10	0.76	8,52	118,04	17801	147.71	0.12	4.05	13.19	107.28
BR4	35378	<0.10	<0.10	19,51	68,94	18744	312.26	0.42	5.03	5.79	42.23
**BR5**	5790	1.72	0.63	1,00	70,82	2041	67.12	<0.10	1.81	5.20	115.56
CV1	2830	<0.10	<0.10	3,50	16,74	9267	52.68	<0.10	<0.10	<0.50	14.75
**CV2**	2119	0.6	0.67	1,67	46,88	2977	50.56	<0.10	0.46	3.50	53.39
**CV3**	1535	1.38	0.87	1,03	56,55	1135	47.96	<0.10	0.80	4.75	61.61
**CV4**	3311	<0.10	0.21	2,85	25,6	9483	57.21	<0.10	0.10	<0.50	21.85
CV5	2909	<0.10	<0.10	4,67	13,11	8979	73.75	<0.10	<0.10	<0.50	12.83
CV6	3534	<0.10	<0.10	3,17	17,96	8510	48.19	<0.10	0.14	<0.50	12.90
**CV7**	3782	<0.10	0.67	2,53	38,67	6124	51.30	<0.10	0.62	1.22	53.46
CV8	2577	<0.10	<0.10	3,22	16,51	7566	51.82	<0.10	<0.10	<0.50	11.89
CV9	3055	<0.10	<0.10	2,97	16,09	8408	49.68	<0.10	<0.10	<0.50	14.09
CV10	3314	<0.10	<0.10	5,42	15,25	9874	90.38	<0.10	<0.10	<0.50	17.10
CV11	2559	<0.10	<0.10	3,76	15,43	8935	52.15	0.19	<0.10	<0.50	11.92
CV12	2648	<0.10	<0.10	3,75	16,07	10350	54.71	<0.10	<0.10	<0.50	11.42
MB1	3236	<0.10	0.17	4,20	13,45	8720	78.55	<0.10	<0.10	<0.50	21.81
MB2	3183	<0.10	0.13	3,92	13,54	9066	73.56	<0.10	<0.10	<0.50	20.95
**MB3**	2924	<0.10	0.39	3,71	28,85	8059	77.89	<0.10	0.69	0.69	38.52
MB4	2587	0.4	0.12	2,67	21,21	9638	47.90	0.24	<0.10	<0.50	14.10
**MB5**	3381	0.18	0.58	2,54	38,71	3728	77.89	0.12	0.82	2.70	45.53
MB6	2505	<0.10	<0.10	4,19	10,98	9386	71.97	<0.10	<0.10	<0.50	13.17
PM1	4424	1.76	<0.10	7,64	15,17	14636	148.27	0.15	<0.10	<0.50	22.64
**PM2**	3128	1.72	<0.10	5,57	25,23	13154	70.98	<0.10	<0.10	<0.50	21.92
PM3	5787	1.65	<0.10	8,79	16,52	16883	162.55	<0.10	<0.10	<0.50	21.48
PE1	6586	<0.10	<0.10	3,62	26,53	12052	62.71	<0.10	0.43	<0.50	14.76
**PE2**	2822	2.52	1,00	2,35	60,18	2017	61.40	<0.10	0.94	5.30	65.92
PE3	4107	<0.10	<0.10	7,25	13,76	13684	147.13	0.15	<0.10	<0.50	21.71
**PE4**	4935	3.11	1.66	2,46	104,7	2162	105.70	<0.10	1.99	10.55	154.03
**CC1**	7071	<0.10	1.93	<0,50	94,86	2736	65.78	<0.10	20.54	3.33	259.88
**CC2**	1505	3.56	2.38	<0,50	150,44	1542	102.25	<0.10	1.68	12.77	373.75
**CU1**	1240	2.59	2.4	<0,50	230,29	1041	106.25	<0.10	0.95	13.62	373.46
**CU2**	21925	<0.10	1.29	1,60	116,1	17785	174.06	0.49	87.55	3.51	206.38
**RO1**	45651	<0.10	1.27	3,71	98,72	21212	126.95	0.20	13.63	1.94	144.51
**RO2**	6800	1.33	2.5	<0,50	173,47	2454	112.46	<0.10	2.93	10.79	275.89

**Fig 2:**
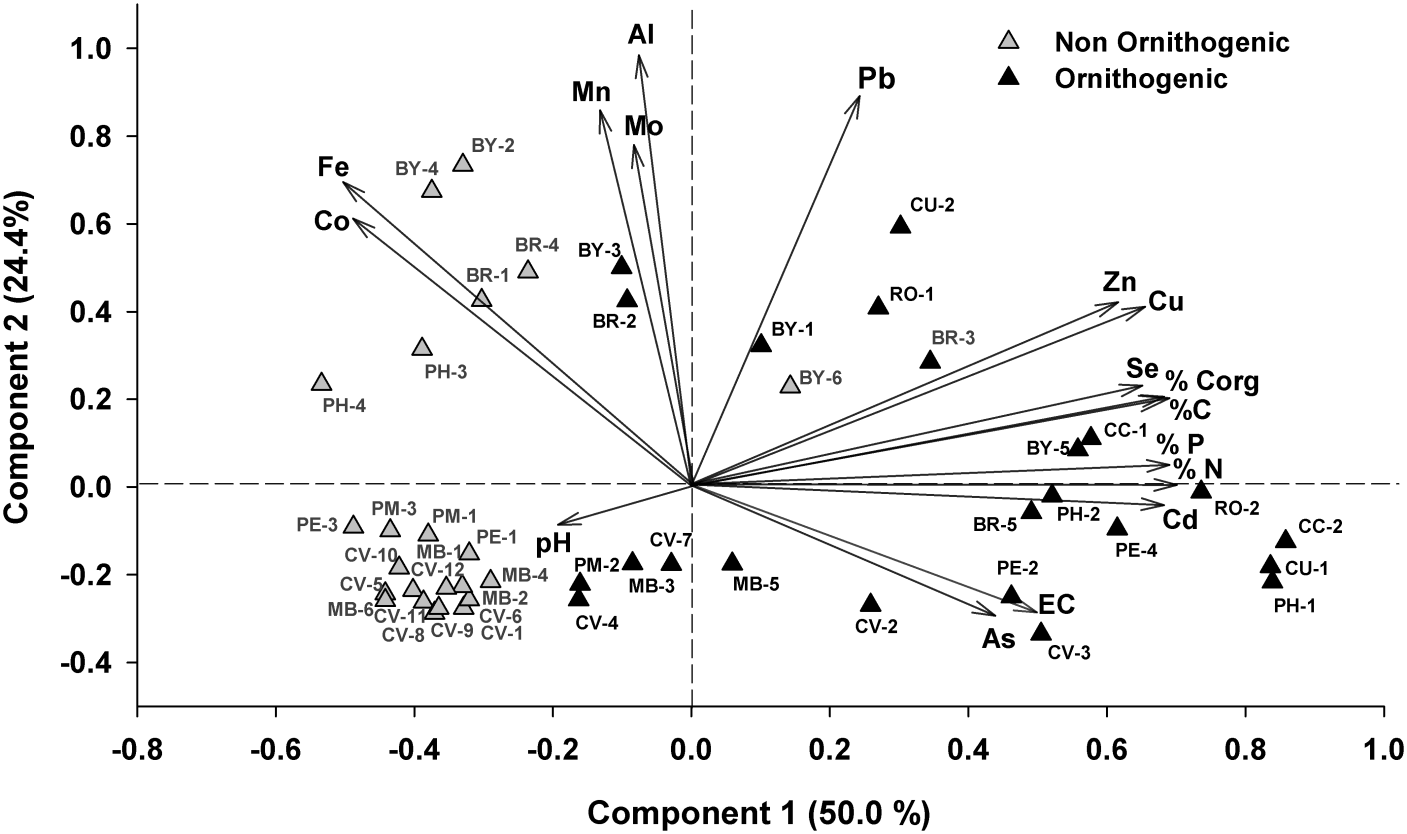
Principal component analysis using the physical-chemical variables and element concentrations of all soil samples (n = 46). Samples are plotted as triangles showing the sample code (see Table 1).

Fig 2 shows a principal component analysis for the physical variables and the most significant chemical element concentrations of all soil samples. The two first PCA axes accounted for 74.4% of the variance underlying explanations on the correlations among the variables. Variables showing the highest positive scores on axis 1 are some of the most explanatory as describing the organic content (%Corg, %C, %N and % P), in addition to the concentration of certain chemical elements (As, Cd, Cu, Se, and Zn). The positive side of axis 2 and the negative side of axis 1 are related to higher relative contribution of elements considered as markers of geochemical origin, mainly Al, Fe, Mn and Mo. All samples coming from non-ornithogenic soils are concentrated in the negative side of the axis 1, suggesting the existence of clear differences compared to samples from sites occupied by rookeries, which concentrate on the positive side of this axis. The only exception is sample BY6, which corresponds to a track frequently used by penguins and elephant seals, which has certain similarities with ornithogenic soils due to the eutrophication produced by the excreta of both species. Therefore, ornithogenic soils are characterized by a high content of Corg, N, P, Cd, Cu, Se, Zn, and for most cases As, meanwhile in non-ornithogenic soils the geochemical elements (Al, Fe, Mn, Mo), when differing from ornithogenic soils, have commonly a greater relative importance compared to the most penguin-affected soils.

**Fig 3:**
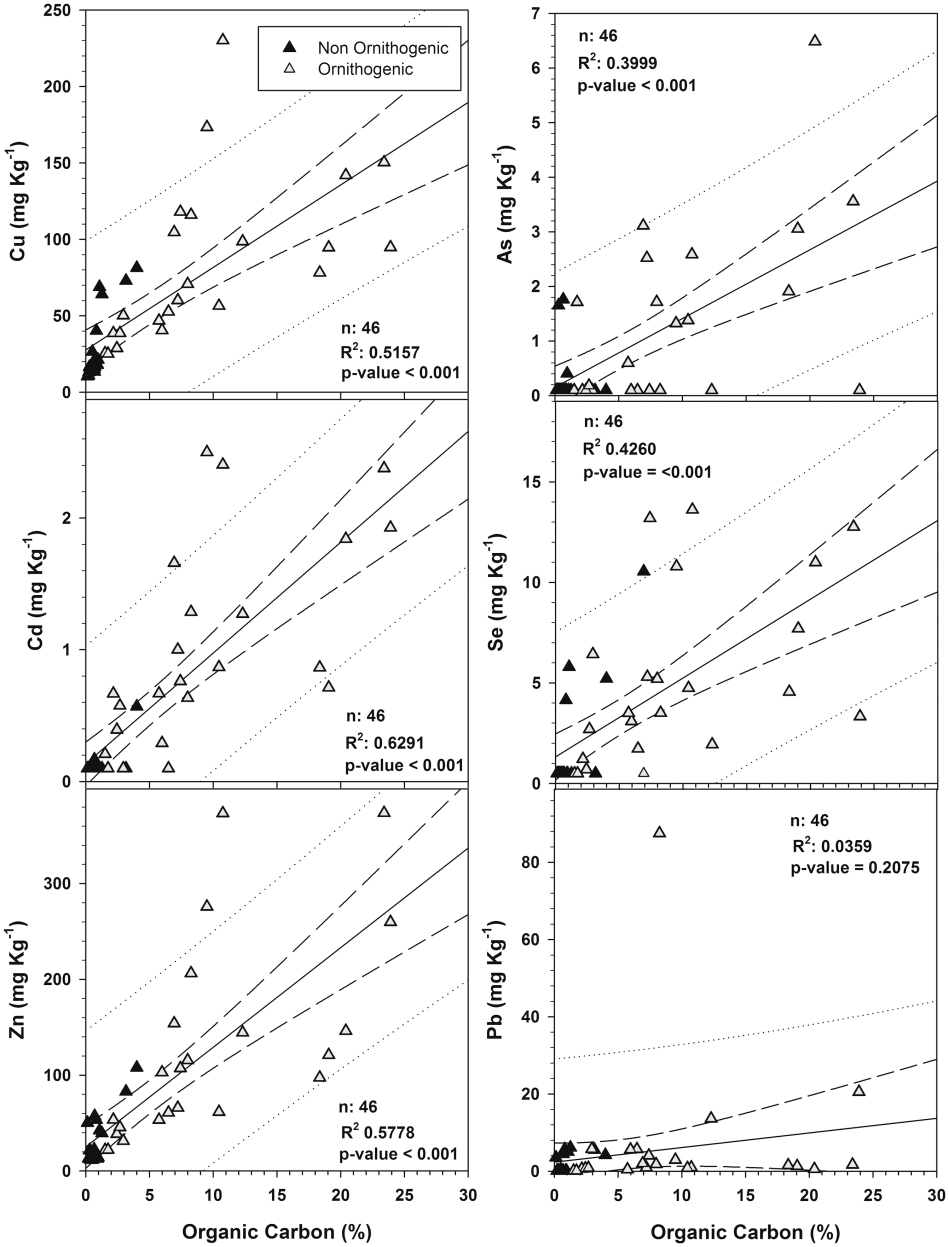
Bivariate correlations between As, Cu, Cd, Pb, Se and Zn, and the % of organic carbon in the analysed soil samples. Note the difference in the scale of concentrations.

These relationships can be more precisely observed when element concentrations are plotted against the % organic carbon (%Corg) in soil, which is the most indicative variable related to penguin influence (Fig 3). Bivariated correlations showing strong statistically significant positive correlations (n = 46, p<0.001) with %Corg were found for As, Cu, Cd, Se and Zn. Contrastingly, although lead concentration increased with organic carbon in some samples, this correlation was not statistically significant (p = 0.207).

To further check whether there were statistically significant differences between the two types of soils considered, two statistical tests (the median test and the Kruskal-Wallis one-way analysis of variance) were additionally performed by grouping the samples separately as ornithogenic and non-ornithogenic soils, respectively. Both tests identified significantly higher values for soil rookeries for EC, %Corg, %C, %N, %P, As, Cd, Cu, Pb, Se and Zn. By contrast, the concentrations of Co and Fe were significantly lower in these soils than in the control areas according to the Kruskal-Wallis (for both elements) and the median (only for Co) tests. Consistent with these results, comparison of elements concentrations inside and outside the rookeries (Table 4) showed that background levels (those of the non-ornithogenic soils) clearly exceeded in most cases in ornithogenic soils for Cd, Cu and Zn. Focusing on the different study sites separately, we observed that the rookery located in Byers Peninsula (BY), far from ship routes (Fig 1), showed heavy metal concentrations within the background range. The opposite occurred in Hannah Point (PH), where all the elements reach values significantly above (As, Cd, Cu, Se and Zn) or below (Al, Co, Fe, Mn, Mo and, Pb) the reference levels. In Barrientos Island (BR), the high concentration of Cd and Zn, as well as the low abundance of Mo, are also remarkable. Soils from rookeries in Deception Island had generally higher levels of Cd, Cu and Zn than their non-ornithogenic counterparts, though the only sample from the outer rookery located in Macaroni Point (PM) did not follow this pattern. More lead also appears on Deception island rookeries except in Macaroni Point. In any case data for Macaroni Point are less illustrative due to the scarcity of samples (n = 1 for the ornithogenic soil). The most polluted soils of Deception Island rookeries are those of Entrance Point, with concentrations over the background range for As, Cd, Cu, Pb Se and Zn. This penguin rookery is totally located in the inner bay of Deception island, in the area with the closest and heavier ship transit among all the studied sites of this island. Comparatively, all samples from the three rookeries located in the Antarctic Peninsula, Cierva Cove (CC), Cuverville Island (CU) and Ronge Island (RO), consistently presented very high concentrations of metallic elements, especially Cd, Cu and Zn, and for most samples also of As, Pb and Se (Table 3).

**Table 4:** Data for rookeries and background levels of metal concentrations (mg Kg^-1^) in the different study sites located in the South Shetland Islands.

		**Al**	**As**	**Cd**	**Co**	**Cu**	**Fe**
Byers Peninsula (BY)	Mean ± Standard deviation, n = 3	37663 ± 6880	1.05 ± 1.42	0.35 ± 0.27	7.17 ± 5.30	62.70 ± 23.20	26055 ± 16199
	Background range	10233–82260	~0.05	0.00–0.71	0.00–68.70	29.39–100.35	0–128229
Hannah Point (PH)	Mean ± Standard deviation, n = 2	**3005 ± 1290 (↓)**	**4.20 ± 2.29 (↑)**	**1.35 ± 0.49 (↑)**	**1.39 ± 0.61 (↓)**	**110.15 ± 31.85 (↑)**	**4183 ± 1661 (↓)**
	Background range	20558–32328	~0.05	~0.05	7.21–9.87	4.99–26.75	37389–38585
Barrientos Island (BR)	Mean ± Standard deviation, n = 3	22542 ± 12830	0.61 ± 0.79	**0.48 ± 0.31 (↑)**	6.38 ± 3.83	79.71 ± 28.37	16040 ± 10783
	Background range	33707–40391	~0.05	~0.05	9.60–22.82	61.77–71.33	13372–34862
Vapour Col (CV)	Mean ± Standard deviation, n = 4	2687 ± 900	0.52 ± 0.54	**0.61 ± 0.24 (↑)**	2.02 ± 0.72	**41.93 ± 11.35 (↑)**	4930 ± 3177
	Background range	2265–3592	~0.05	~0.05	2.24–5.37	13.27–18.52	7357–10615
Baily Head (MB)	Mean ± Standard deviation, n = 2	3153 ± 229	0.12 ±0.07	**0.49 ± 0.10 (↑)**	3.13 ± 0.58	**33.78 ± 4.93 (↑)**	5894 ± 2166
	Background range	2210.40–3545.04	0.01–0.45	0.03–0.20	2.49–5.00	7.11–22.48	8513.42–9891.58
Macaroni Point (PM)	Mean, n = 1	**3128 (↓)**	1.72	0.05	**5.57 (↓)**	**25.23 (↑)**	**13154 (↓)**
	Background range	3743–6469	1.60–1.82	~0.05	7.07–9.37	14.50–17.20	13513–18007
Entrance Point (PE)	Mean ± Standard deviation, n = 2	3879 ± 1057	**2.82 ± 0.30 (↑)**	**1.33 ± 0.33 (↑)**	2.41 ± 0.05	**82.44 ± 22.26 (↑)**	**2090 ± 73 (↓)**
	Background range	2868–7826	~0.05	~0.05	1.81–9.07	7.37–32.92	12052.30–14500
		**Mn**	**Mo**	**Pb**	**Se**	**Zn**	
Byers Peninsula (BY)	Mean ± Standard deviation, n = 3	223.23 ± 144.91	0.17 ± 0.09	4.21 ± 2.05	4.17 ± 2.55	94.92 ± 25.18	
	Background range	0.00–1082.39	0.00–0.69	3.79–6.52	0.00–7.47	36.76–125.90	
Hannah Point (PH)	Mean ± Standard deviation, n = 2	**91.01 ± 18.83 (↓)**	**0.05 ± 0.00 (↓)**	**1.05 ± 0.49 (↓)**	**7.78 ± 3.22 (↑)**	**121.85 ± 24.41 (↑)**	
	Background range	298.32–310.24	0.20–0.22	3.11–4.89	~0.25	46.70–60.84	
Barrientos Island (BR)	Mean ± Standard deviation, n = 3	136.11 ± 52.25	**0.17 ± 0.13 (↓)**	3.86 ± 1.61	8.27 ± 3.51	**84.74 ± 37.88 (↑)**	
	Background range	133.48–371.86	0.38–0.44	5.03–6.65	0.00–8.56	37.82–43.70	
Vapour Col (CV)	Mean ± Standard deviation, n = 4	51.76 ± 3.38	0.05 ± 0.00	**0.50 ± 0.26 (↑)**	**2.43 ± 1.78 (↑)**	**47.58 ± 15.23 (↑)**	
	Background range	31.22–87.12	0.00–0.17	0.00–0.12	~0.25	9.83–16.89	
Baily Head (MB)	Mean ± Standard deviation, n = 2	77.89 ± 0.00	0.09 ± 0.04	**0.76 ± 0.07 (↑)**	**1.70 ± 0.00 (↑)**	**42.03 ± 3.51 (↑)**	
	Background range	44.29–91.70	0.01–0.27	~0.05	~0.25	9.71–25.30	
Macaroni Point (PM)	Mean, n = 1	**70.98 (↓)**	0.05	0.05	0.25	21.92	
	Background range	141.13–169.69	0.00–0.20	~0.05	~0.25	20.90–23.22	
Entrance Point (PE)	Mean ± Standard deviation, n = 2	83.55 ± 22.15	0.05 ± 0.00	**1.47 ± 0.53 (↑)**	**7.93 ± 2.63 (↑)**	**109.98 ± 44.06 (↑)**	
	Background range	20.50–189.34	0.00–0.20	0.00–0.62	~0.25	11.29–25.19	

Metal concentrations for rookeries out from the background range are in bold: (↓) the 95% confidence interval is below the background range; (↑) the 95% confidence interval is above the background range.

**Fig 4:**
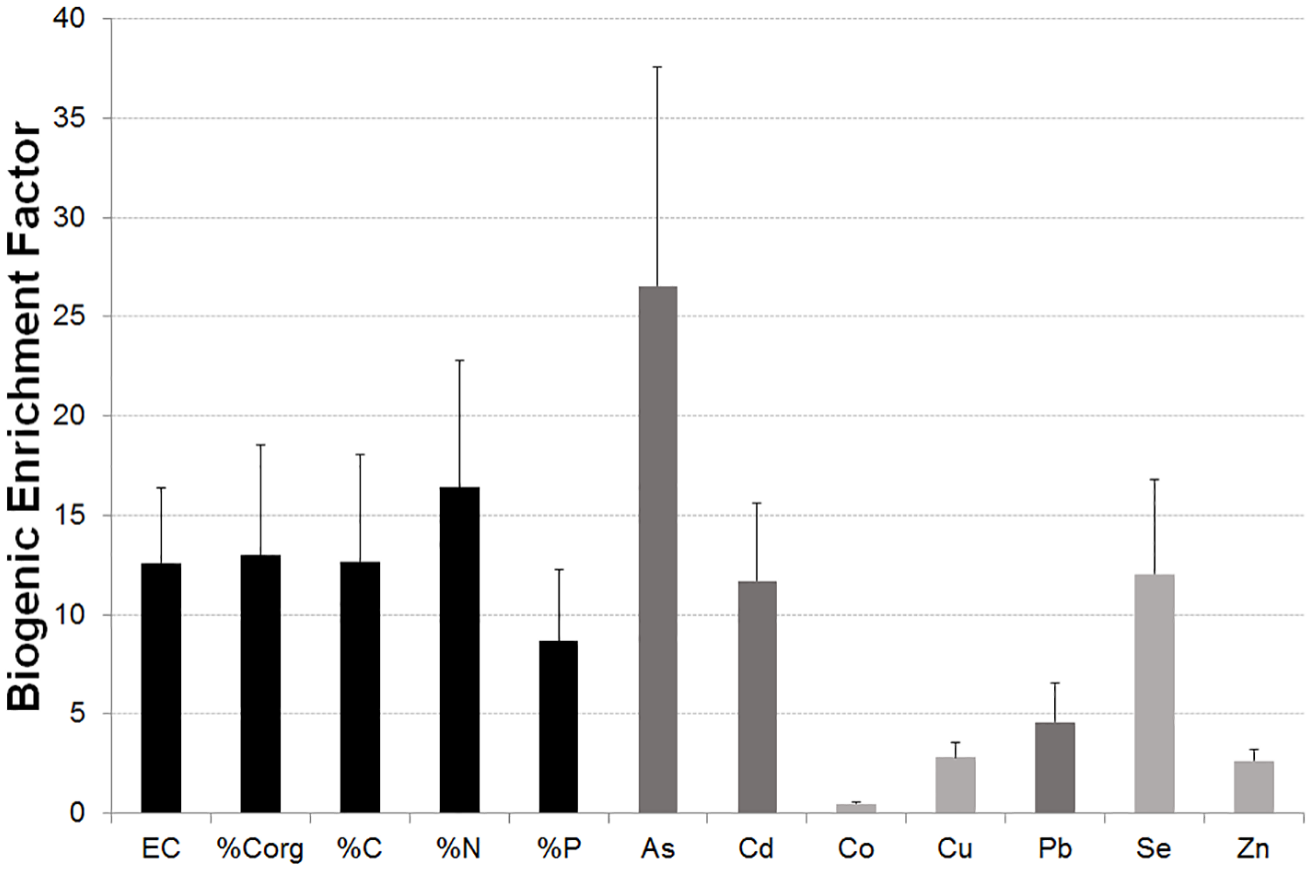
*Biogenic enrichment factor* in the rookeries of basic soil properties (black bars), toxic trace elements (dark grey bars) and essential trace elements (light grey bars). Data are presented as mean ± standard error of the mean. N = 7. Only variables presenting statistically significant differences between ornithogenic soils and non- ornithogenic control areas are shown (Median test, p-value<0.05).

The enrichment for certain elements in ornithogenic soils (BEF, Biogenic Enrichment Factor) can be more clearly seen in Fig 4. Rookeries’ soils have much higher levels of certain macronutrients (C, N, and P) and other elements (As, Cd Cu, Pb, Se, and Zn), indicating that they were the most likely undergoing bioenrichment. This process affected especially to three elements: As (BEF = 26.54), Se (BEF = 12.05) and Cd (BEF = 11.71). The electrical conductivity was also significantly increased in these areas (BEF = 12.59), while Co appeared in significantly lower concentrations in rookeries than in the control areas (BEF = 0.47).

### Influence on element concentrations of geographical and biological factors

Apart of the aforementioned differences between the sites, non-parametric median tests were applied to evaluate whether there was some regional segregation of the samples comparing the soils samples from rookeries of the South Shetland Islands and the Antarctic Peninsula. These tests showed only significant differences (p<0.05) between ornithogenic soils from the South Shetland Islands and the Antarctic Peninsula for %C, %Corg and Cu. Thus, no significant differences for most of the parameters analyzed in this study can be attributed to the regional geographical factor, although certainly the overall penguin impact on the organic content of soils of the rookeries of the Antarctic Peninsula was strongest. Contrastingly, local factors, as shown by the PE site showing the highest pollution in the place nearest to the most intense ship traffic, and the Byers Peninsula and Macaroni Point rookeries, farther from ship routes, displaying the lowest concentrations of pollutants, can be more important. On the other hand, the comparison of Chinstrap and Gentoo penguin colonies from the same locations where both species jointly appeared (Hanna Point and Barrientos Island) did not show differential patterns among these species, as the soil concentrations of some elements where higher in one of the sites for one of the species and lower in the other site. Thus, no consistent pattern was found when both species were considered for the element concentrations in the soils occupied by each species within the same locations.

### Soil microbiota composition

**Fig 5:**
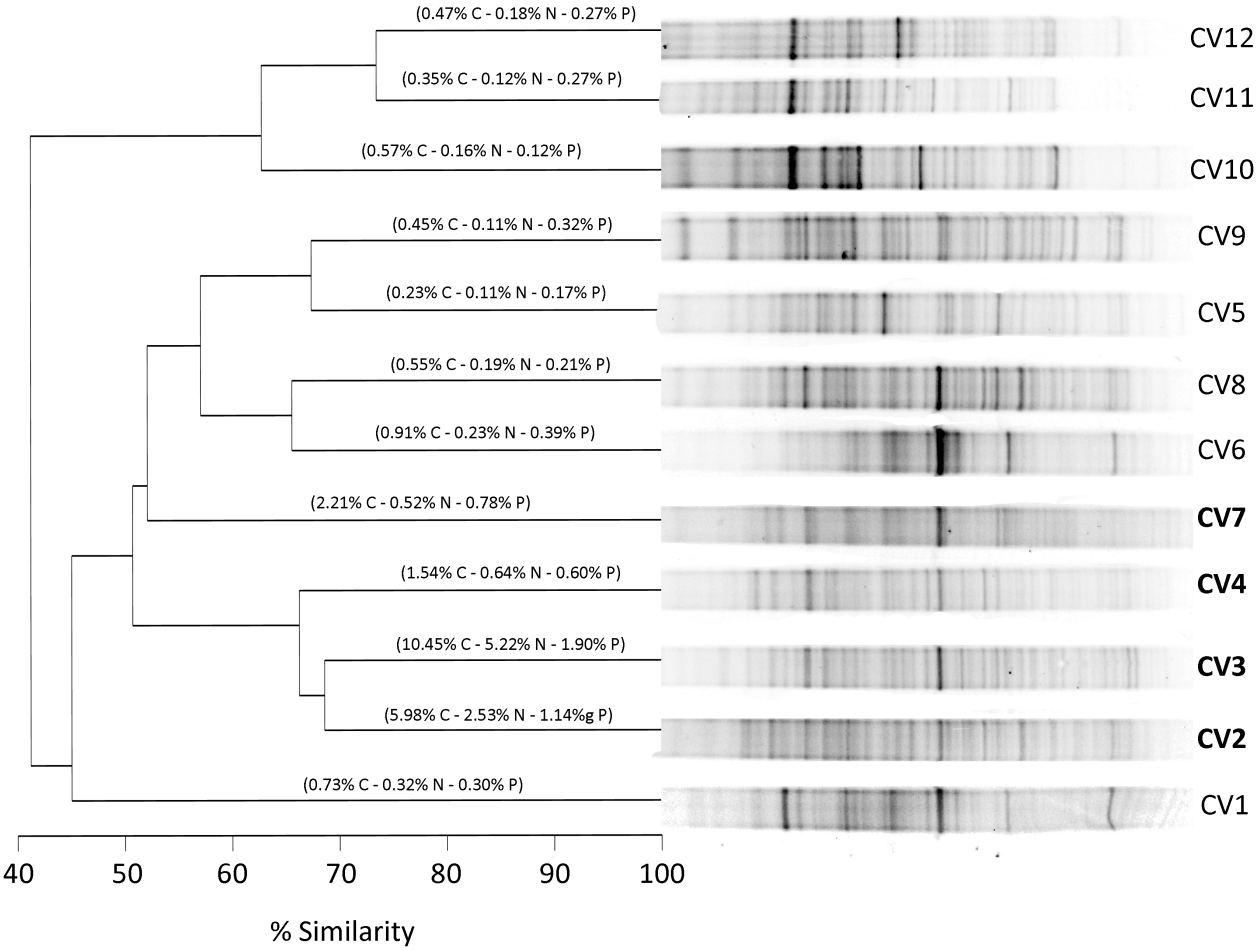
Dendrogram showing the similarity of the electrophoretic band pattern (fingerprinting made by DGGE) of the extracted and PCR amplified DNA encoding for the 16s rRNA gene sequence for samples of Vapour Col, Deception Island. The relative contribution (% dw) of C, N and P to soil weight is shown for each sample to illustrate about penguin influence. In bold, the most penguin affected soils (CV2, CV·, CV4 and CV7), note that they cluster together.

Fig 5 shows the DGGE fingerprints and the dendrogram resulting from the similarity among sample’s diversity pattern of soil microbiota for Vapour Col, which is the most complete site with respect to the number of samples as well as the site better covering the spatial distribution within the rookery because of the concentric sample design. Values of the percent of C, N and P in soil are also given within the plots, to offer an idea of the relative influence of penguins. The similarity analysis shows well the pattern with the most organic-rich soils (CV2, 3, 4 and 7) due to penguin influence appearing as having the most similar dominant microbiota, clustering with the lowest distances, whereas they clearly differ from those of non-ornithogenic soils.

Concerning the phylogenetic affiliation of the main bands sequenced, those that were clearly separated and had high intensity were excised from DGGE gels and were successfully determined by Sanger sequencing, then a sequence similarity search of partial 16S rRNA gene was completed. Sequences of main bands from CV are available at figshare.com, https://doi.org/10.6084/m9.figshare.5212003.v2). In ornithogenic soils, bacterial diversity was much lower than in non-ornithogenic soils. In the former, a very high dominance of the phylum Firmicutes (65% of the bands) was found, two thirds of them belonging to the family Clostridiaceae and a third to the Bacillaceae. The rest of the sequenced bands from ornithogenic soils were affiliated to the Class Deltaproteobacteria (27%), mainly to the family Desulfobacteraceae, as well as to the phylum Bacteroidetes (9%). Although Firmicutes also accounted for an important part (38%) of the sequenced bands also in the non-ornithogenic areas (especially in the margins of the colonies were there was still some penguin affection), typical soil Actinobacteria, all from the family Actinomycetales, were also co-dominant (32% of the sequenced bands). In these non-ornithogenic soils Alphaproteobacteria of the family Rhodobacteraceae were also abundant (18% of the bands), and other taxa (Chloroflexi, Nitrospira, Gammaproteobacteria) also appeared to a lesser extent.

## Discussion

### Penguins as biotransporters of nutrients and metals

Because of their ability to deliver nutrients from sea to land and to enhance the availability of local resources, seabirds have been the focus of many studies of nutrient transfer [49]. Previous studies hypothesised that penguin excreta could be a local weak contaminant for soil [17,23]. Our study provides evidences to the insights of other recent papers where the increase of certain element concentrations in ice-free Antarctic soils due to penguin influence is suggested [30,50]. In addition to demonstrating that penguins act as metal biotransporters, our study has identified, a) which elements are increased to higher concentrations in the rookeries, b) in which sites this increase has exceeded the background levels generating biogenic pollution, c) what are the differences existing between the two analyzed penguin species, and d) what consequences this enrichment has for the soil microbiota.

### Elements accumulated in rookeries

Even though ICP analyses allowed for the sequential determination of many elements, among those elements analysed our focus was posed on the concentrations of potential harmful elements that can be associated to anthropogenic pollution, mainly As, Cd, Cu, Pb and, although they can be considered as micronutrients, also Zn and Se. In addition, other metals that are commonly associated with geochemical processes, mainly Al, Fe, and Mn, were also studied. As penguin activity increases the organic content of soils, mainly by faecal inputs, the multivariate correlation of variables related to organic matter content (%C, %N, %P, %Corg,), positively with elements coming from anthropogenic pollution or negatively with those of geochemical origin, illustrates to what extent the observed concentrations of different elements are linked to penguin activity. This first approach allows assessing the effect of biotransport into soils by these animals. The general pattern found demonstrated that the soil samples more affected by penguin deposition, detected by their higher organic contents, show the highest concentrations of the metals associated with anthropogenic pollution. Even though their concentrations are not extremely high, especially when compared to polluted sites or to baseline concentrations of standard soils [51], they over exceed by several times the base levels of penguin-unaffected soil samples studied (Fig 4 and Table 4). This supports our hypothesis that penguins are transporting pollutants from sea to land, including some toxic metals such as those reported.

Regarding metals and metalloids, other studies [3] identified Cu, Pb, Zn, Cd, As and Hg as the most common in Antarctica. Some of these elements can have an anthropogenic origin. Amaro et al. [13] and Padeiro et al. [22] pointed out the existence of several contamination hotspots on the Fildes Peninsula, King George Island, a place characterized by a high density of scientific stations and a long human occupancy. These highly polluted sites were mainly related to high levels of Pb and Cd (samples near fuel tanks), Cr and Ni (samples near waste disposal sites), or Zn (both fuel tanks and waste disposal sites). On the other hand, certain trace elements have been recorded in rookeries. For example, Espejo et al. [17] found high levels of Pb, Cu and Zn in excrements of two species of penguin from different locations of the Antarctic Peninsula; Smichowski et al [9] studied the content of trace elements in penguins from King George Island and; Huang et al [50] demonstrate that the emperor penguin can transport a large amount of nutrients and contaminants from ocean to land even with a relatively small population. Consistently with these studies, our research detected As, Cd, Se, Cu, Pb and Zn in relatively high concentrations in penguin-affected soils.

Arsenic is a non-essential element that is considered to be an environmental contaminant because of its bioaccumulative capacity and global occurrence. The Antarctic Peninsula and surrounding islands exhibit high volcanic activity, which could explain the presence of arsenic in the region [9]. Depending on its oxidation status, arsenic can be dissolved in water, from which it can be absorbed by algae and krill, then entering the food web, passing to predators such as penguins, and consequently being relatively abundant in penguin guano [23]. However, in our samples As values are in the same level of magnitude as the baseline concentrations proposed for different Antarctic sites [13,52,53] and far away for the extreme values obtained in sites with a distinctive magmatic evolution as Murature Beach, in Deception Island [11]. Arsenic concentrations recorded in highly contaminated sites in Fildes Peninsula by Padeiro et al. [22] were also greater than our data. Therefore, As levels in the analyzed rookeries do not seem to be generally and clearly increased regarding background levels of the site and can be mostly attributed to natural sources, although some average values are high (Table 4).

Cadmium is a toxic metal coming both from natural and anthropogenic sources, which is known to bioaccumulate by the marine biota. It binds strongly to metallothioneins, proteins in the membranes of cell organelles, in the kidney of marine vertebrates, and levels increase with age in some marine mammals [9]. Additionally to anthropogenic sources, upwelling of Cd-rich waters and local volcanism can be two important sources for a natural enrichment of Cd in polar food webs [54]. Grotti et al. [18] also demonstrated its incorporation in the Antarctic biota at different trophic levels, including the krill, which is the primary food source for penguins. Recently, Jerez et al. [19] showed that accumulation and magnification of several elements by penguins can be occurring within our study area, and reported Cd, as we do, as the most relevant heavy metal being accumulated by penguins, in such a way that Cd reached levels potentially toxic in some specimens. Cd concentrations in rookeries exceeded background levels in most cases, suggesting that the biotransport of Cd by penguins from sea to land is significant. This increased exposure to Cd in penguins could explain the observed high selenium level, since Se is known to have a detoxifying effect on this heavy metal [9]. Cooper is also present at high levels in Antarctic krill [19], which can also be related with the high levels detected in soil rookeries resulting from penguins feeding on these crustaceans.

Copper is abundant in sea water and its origin is associated with natural sources such as diffusive fluxes, upwelling and continental weathering [11]. We found high concentrations of this metal with respect to the background values, which could point to a significant transport by penguins. However, the mean values reached in these areas are comparable to background values recorded in other Antarctic zones [11,13]. These concentrations are also below those values recorded in areas contaminated by human activities in the Fildes Peninsula [13] or in Ross Island [55].

Lead can mostly originate from different human sources, such as fuel combustion, waste incineration, sewage disposal, paints or accidental oil spills [6,8,30]. Therefore, human activity can increase its local concentrations in soils over the natural background, as was demonstrate by Padeiro et al [22]. On the other hand, lead isotopic composition in water samples collected in the Weddell Sea suggested that the cycling of Pb has been influenced by industrial activities from South America [56], showing the anthropogenic influence of human activity in the concentration of this metal in sea waters. However, we do not always found a general increase of Pb concentrations in soils associated to penguin activity. Certainly, lead exceeds background levels in three of the seven sites shown in Table 4, however in Hannah Point the concentration of this element in the colonies of both penguin species is was lower than that of the surrounding soils. This metal has a strong affinity to bones, hair, nails, feathers and claws [23] and, consistently, penguin excreta usually present a low concentration of Pb [16]. This could explain why Pb is not always abundant in rookeries' soils.

Zinc, like cooper, is an essential element to birds, and vital metabolic processes probably mainly regulate the concentrations [21]. This metal follows the same trend that Cd, with high values in rookeries in comparison with analyzed near soils. We obtained concentrations above the background levels, but much lesser than those recorded in polluted sites close to Antarctic research stations [13], or areas used during the 'Heroic Age' of Antarctic exploration [55]. Therefore, Zn concentration may be higher than expected because of the penguins’ activity, but without generating strong local contaminations.

Summarizing, as the PCA and other analyses shows, the association of elements with factors can be indicated by natural (geogenic and pedogenic characteristics) and animal influences (BEF). The differences found between both groups of elements can be attributed to the fact that some, such as Cd, Cu, Se and Zn, are somewhat influenced by the exogenous contamination (biogenic enrichment factor due to penguins rockeries) whereas Al, Co, Fe, Mn and Mo would be dependent of the parent rock contents. The highest Fe, Mn concentrations are found in ultrabasic igneous rocks, followed by basic rocks (gabbro and basalt), whereas sedimentary rocks are especially poor in As, Se and heavy metals.

### The significance of background levels

The analysis of the concentrations of metals and other pollutants requires background information for each particular site, since soil parameters, including the concentrations of different chemical elements, can vary significantly at relatively small spatial scales, especially in volcanic areas as those widely spread over the maritime Antarctic. Sampling in sites next to the penguin colonies but lowly affected by animal influence is thus needed in order to establish these background levels. For this task, we consider as more accurate the use of the background range (Table 4) rather than that of BEF (Fig 4), since the mean values of BEF may mask some important differences. However, both were calculated.

Background levels can be markedly affected by local geology. For example, Deception Island is an active volcano where four colonies of penguins have been studied, all of them showing soil metals concentrations that increased with penguin activity. Deheyn et al. [20] have suggested that geothermal activities enhanced the bioavailability of trace element pollutants in Deception Island, which could explain its higher trace metals levels compared to other areas of the maritime Antarctica. Our results, however, show different levels of bioaccumulation depending on the situation of the penguin rookeries, with higher concentrations of pollutants in the rookery located in the inner bay of this island, Entrance Point. There, As, Cd, Cu, Pb, Se and Zn concentrations are over the reference level. Penguins of this colony frequently fed in the inland waters of Port Forster. This inner bay is more polluted than the outdoor areas due to its poor rate of water exchange (1% volume exchange over each tidal cycle, [57]) in combination with a high ship navigation during austral summer mainly linked to tourism activities, with a six-fold increase by in the last 2 decades [17,35,58]. Comparatively, however, soils from Macaroni Point, the outer rookery of Deception Island being slightly less affected by ship transit, show lower pollutant concentrations compared to the other rookeries from this island. However, only one soil sample from a penguin colony was retrieved for Macaroni Point, and therefore these results must be interpreted carefully. Probably, for Deception Island, a combination of natural and anthropogenic inputs explains the biotransport of trace elements by penguins and its accumulation in soils.

The influence of ship traffic, both by touristic and scientific activities, could also be a complementary factor that would be affecting biotransport of trace metals by penguins in other studied locations. This could be the reason why samples from Cuverville and Ronge Islands have the highest concentrations for most of the analyzed metals. Both sites are located in the Herrera Channel, a mandatory route for most of tourist ships visiting this area [34]. A similar situation could occur in Hannah Point, other of our study sites where elements such as As, Cd, Cu, Pb, Se and Zn are also in high concentrations. Contrastingly, the rookery of Byers Peninsula, where concentrations of the studied elements were similar to the background levels, is the farthest from ship routes among those studied. Byers Peninsula is a nearly pristine area [59,60] presenting biological communities lowly impacted by direct anthropogenic impacts [61]. On the other hand, the highest organic enrichment and metal content in soils of rookeries of the Antarctic Peninsula could also be enhanced by a concentration effect, since there the ice-free available land is relatively lower than in the South Shetland Islands, and the penguin impact of penguins per unit area could even be stronger. In any case, our data suggest an association between ship traffic and the increase of some metals in soils of the nearby penguin colonies. A part of this pollution could also come from the chemical legacy produced by human activity prior to the entry into force of the Protocol on Environmental Protection in 1998 [5].

### Differences between penguin species

We failed to detect differential trends in biotransport of metals and metalloids from sea to land for the two studied *Pygoscelis* species. In principle, the concentrations of these elements in soil rookeries could vary depending on the diet and internal needs of penguins. About 15–40% of the diet of Gentoo penguins is composed by benthopelagic fish and small squids, and the rest of its diet is based on krill and different crustaceans [62]. Meanwhile, Chinstrap penguins eat mainly krill and crustaceans [63]. Although a more diverse diet could be the reason for a bioaccumulation of a more wide range of metals by Gentoo penguin, as observed in previous studies developed on feathers [16], we could not demonstrated any differential metal biotransport among the two studied species to the rookeries’ soils. As in our study, Espejo et al. [17] did not found a very clear pattern on differences of metal content when comparing these two penguin species, which, compared to the study of Jerez et al. [16] suggest that the pattern of metal bioaccumulation and excretion could differ. Even if differential bioaccumulation could exist between these two penguins’ species, the increasing of elementary concentrations in ornithogenic soils depends not only on this bioaccumulation, but also on the dropping through penguin excrements.

### Consequences for soil microbiota in the rookeries

Concerning the composition of the soil microbiota, we performed a molecular study of soil samples collected in 4 different areas (Chinstrap penguin rookeries) from Deception Island, CV, MB, PE and PM. For samples obtained from the concentric sampling in CV, which spatially covers well the penguin rookery, the results of the bands (OTUs) pattern are shown in the dendrogram of Fig 5. There, clustering by the microbial community fingerprinting coincides with that given by the relative abundance of C, N and P, as markers of penguin affection of soils, showing that penguin influence also shapes the composition of the soil microbial community.

It is widely accepted that the intensity of each DGGE band can be directly related to the relative abundance of the population of each bacterial taxon, so that we can also relate the relative intensity of the different bands, once sequenced, with penguin influence on the shaping of the soil bacterial community. We found many DGGE bands related to microorganisms typical from faecal contamination in all studied sites but, especially, in ornithogenic soils, mainly those of the phyla Firmicutes (Clostridiaceae and Bacillaceae) and Bacteroidetes. Apart of these dominant faecal bacteria, the relative frequent appearance of sulphate-reducers from the family Desulfobacteraceae (Deltaproteobacteria) in these soils could be favoured by the anaerobic conditions induced in parts of the soil due to the very high oxygen demand originated by the excess of organic matter caused by massive penguin excreta. Contrastingly, in non-ornithogenic soils, even though they are also somewhat affected by penguin dropping as being in marginal areas of the rookeries, faecal bacteria share their dominance with a much more diverse soil community dominated by typical soil Actinobacteria (Actinomycetales). Alphaproteobacteria of the family Rhodobacteraceae, typically featured as aquatic bacteria that frequently thrive in marine environments [64], are also relatively abundant and could be easily brought by the sea splash heavily reaching the colonies in these highly windy areas. Very recently, the first description of the Chinstrap penguin gastrointestinal tract microbiota through pyrosequencing, which was conducted in the same Vapour Col penguin rookery that we studied, was published [65]. It demonstrated that Firmicutes (58% of the reads), mostly Clostridia and, to a lesser extent also Bacilli, as well as Bacteroidetes (16.6%), and Proteobacteria (9.7%), the later mainly Beta- Gamma- and Epsilonproteobacteria, were dominant in cloacal samples of Chinstrap penguin. Although these authors found large differences between chicks (which had more Firmicutes) and adults (with more Bacteroidetes and Proteobacteria), representative taxa of these phyla were consistently found as dominants in the penguin gastrointestinal microbiota. This study also found that there were large differences, though not so at the phylum level, in bacterial community composition Chinstrap penguins compared to other Antarctic penguins, including the congeneric Adélie and Gentoo penguins. These results of Barbosa et al. [65] show the genuine faecal bacterial community of Chinstrap penguins, and its composition is quite similar in the dominance of the main taxa to that of the ornithogenic soils we studied. Contrastingly, our study shows that the non-ornithogenic soils of the marginal areas of the Chinstrap penguin rookeries, even they are somewhat affected by penguin activity, show a much more diverse bacterial community in which typical soil. Actinobacteria (10-fold more abundant in the non-ornithogenic soils compared to penguin cloacal samples) or even Alphaproteobacteria (100-fold more abundant in our non-ornithogenic than in the penguin faeces) account for most of the bacterial community in non-ornithogenic soils. This shows the strong influence of penguin drooping on the relative bacterial abundance, shaping the bacterial soil community.

Although we have demonstrated that the areas most influenced by penguins also present higher concentrations of the biotransported elements, it is likely that the main changes promoted in the soil microbiota are determined by the extraordinary supply of faecal bacteria with penguins excrements, which totally alters the relative abundance of soil bacterial taxa within the community, but cannot be specifically attributed to increased organic or metal content but instead to direct dropping with penguin faeces.

These ornithogenic soils enriched with guano-derived compounds generally present low C/N ratio values [66, 67], which may be due to the presence of high levels of inorganic N forms [68]. Some observed very low C/N ratios in analysed soil samples also suggest different rates of transformation of the organic matter and mineral nitrogen in the guano deposits by soil microbiota [66].

## Conclusions

Our current findings demonstrate that penguins do transfer organic and inorganic nutrients, metals, and other elements, mainly As, Cd, Cu, Se, and Zn, from the sea to the terrestrial ecosystem, being an important pathway system for pollutant transport to Antarctic coastal areas. Natural geochemical processes can enhance trace elements bioavailability in certain volcanic areas of the maritime Antarctica, but human influence by ships transit and other local (directly) -or global (through long distance transport) activities could also contribute to increase the levels of these pollutants. This may have implications for any terrestrial biota, due to the entrance of these elements into the terrestrial food webs. However, we did not find a strong influence affecting the composition of the soil microbial community as consequence of specific metal enrichment. Soil microbiota is strongly altered by faecal inputs of penguins, but nevertheless a base pool of common soil bacterial species remains among the principal members of the community when soils are only slightly affected, turning into an absolute dominance of faecal bacteria in the most affected areas of the colonies. The enrichment could, however, additionally affect the functioning of other living organisms (e.g. native bryophytes, lichens and vascular plants, soil invertebrate fauna, etc.) including penguins [30,31] and/or locally connected ecosystems (lakes, ponds, and streams). This issue deserves further investigations. Cumulative deposition of metals and nutrient enrichment by penguins could contribute to create alterations of biological productivity in the naturally nutrient-poor soils of Antarctica, but also could generate other impacts such as the establishment of cosmopolitan species or the decrease of biodiversity by the spread of a few organisms best adapted to eutrophic environments, with concentrations of potentially toxic elements much surpassing the background levels. An exhaustive pedological study and assessing of available concentration of harmful elements in soil environment, especially for As, Cd, Cu, Se and Zn, are needed in the future to determine ecological and ecotoxicological risks.

The Antarctic environment is often regarded to be pristine and unpolluted, and so one might expect low to very low levels of compounds associated with environmental pollution. However, our research has shown that the levels of metals and other potentially toxic elements may be locally high in soils due to biotransport of penguins from sea to land. Given the natural abundance of penguin colonies at coastal ice free sites this effect may not be negligible. Penguins' role in the biogeochemical cycle between ocean and land should be further explored, especially in the Antarctic land systems, which are generally characterized by a low-nutrient content. It is necessary to investigate now baseline levels of chemical elements to assess possible future changes in Antarctica within long-term monitoring programs. Our research also provides useful information about the levels of the metallic elements both in soils affected and unaffected by penguin rookeries and, more important, it highlights that even in the supposedly more pristine environments anthropogenic impacts could be magnified by the interaction with the autochthonous biota, thus influencing fluxes within the biogeochemical cycles. Antarctic biological communities are experiencing increased levels of human pressure and pollution. In this regard, the ecotoxicological effects could become more prominent as geographic isolation and environmental harshness had maintained them disconnected from human pressures and impacts, so that they may have limited tolerance and may not show resilience in front of these new increasing pressures.

## Supporting information

S1 Table Brief description of sampling sites. (DOCX)

S2 Table Standard element recovery (%) comparison for methods used in our study, to those given by the method 3050B given by USEPA [38] and those used by Roca-Perez et al. [39]. (DOCX)

S3 Table Raw data for soil variables given in Table 2. R stands for each replicate.
(DOCX)
