# Supplementary material for: Soil features in rookeries of Antarctic penguins reveal sea to land biotransport of chemical pollutants
Source: PLoS One. 2017 Aug 16;12(8):e0181901. doi: 10.1371/journal.pone.0181901 (PMC5558944; doi:10.1371/journal.pone.0181901)
Supplement: S1 Table — (DOCX) [file pone.0181901.s001.docx]

**S1 Table.** Brief description of sampling sites.

| **Area** | **Site** | **Description** |
| --- | --- | --- |
| South Shetland Islands | Livingston Island: Byers Peninsula (BY) and Hannah Point (PH) | This island holds two sites with colonies surveyed: Hannah Pt. and Byers Peninsula (Devil´s Point). Both rookeries are predominantly constituted of Gentoo penguins (ca. of 6,000 and 15,000 breeding pairs respectively), but with sparsely chinstrap's in Byers (roughly 200) and a small rookery of this last species in Hannah Point (approximately 1000 pairs). Byers Peninsula is a protected area (ASPA 126) deglaciated through the Holocene [32,33], which rarely receives visitors (only scientists), whereas Hannah Point is an important tourist destination with up to 5,000 visitors per year. It is situated in the main route of tourist cruises, even if some of they might not necessarily disembark there [34]. The closest research station ‘Juan Carlos I’ is 30 km from Hannah Point. |
|  | Barrientos Island (BR) | This islet sustains colonies of both penguin species (c.a. of 5,000 Gentoo's breeding pairs, and roughly 2000 of chinstrap's). It is a remarkable visited site by tourists with an average of 7,000 visitors approximately on the last years according to IAATO.org website [35]. The Ecuadorian station ‘Maldonado’ is present nearby (5 km). |
|  | Deception Island: Vapour Col. (CV), Baily Head (MB), Macaroni Pt (PM), Entrance Pt (PE) | This island is an active volcano with four colonies of Chinstrap penguin: Vapour Col. (c.a. of 30,000 breeding pairs), Entrance Pt. (roughly 1,000 pairs), Baily Head (up to 90,000 pairs) and Macaroni Pt. (roughly 1,000 pairs). The inner waters of the island hold two scientific stations (the Spanish Gabriel de Castilla and the Argentinian Decepción) and four visit sites, which represents an intense human activity, particularly regarding the steady flow of ships, being the most exposed colony that of Entrance Pt while the rest of the colonies are facing open waters. Baily Head receives a moderate amount of visitors annually (ranging from 1,000 to 5,000) whereas Vapour col. receives scientific visitors only but rather frequently. Lastly, Macaroni Pt has rarely any visitors at all. |
| Antarctic Peninsula | Cierva Cove (CC) | This site, located in Danco Coast, contains a Gentoo penguin colony of medium size (roughly 5,000 breeding pairs). This colony is beside a scientific station of low occupancy (the Argentinean Station Primavera). |
|  | Cuverville Island (CU) and Ronge Island (RO) | This couple of islands is located side to side in Graham coast, containing respectively colonies of Gentoo penguin (c.a of 5,000 breeding pairs in Cuverville and roughly 1,000 pairs or less in Ronge). Cuverville site has a lot of tourist visits (more than 10,000 per year) being one of the top 10 in Antarctica, whereas Ronge is theoretically rarely visited, and only by scientists. These colonies are facing each other at a very close distance (separated by no more than a few hundred meters) thus likely sharing the same fishing waters. The waters between them are a typical route for tourist cruises, as well as for independent yachts or even scientific vessels [34]. |
