# Supplementary material for: Soil features in rookeries of Antarctic penguins reveal sea to land biotransport of chemical pollutants
Source: PLoS One. 2017 Aug 16;12(8):e0181901. doi: 10.1371/journal.pone.0181901 (PMC5558944; doi:10.1371/journal.pone.0181901)
Supplement: S2 Table — (DOCX) [file pone.0181901.s002.docx]

**S2 Table.** Standard element recovery (%) comparison for methods used in our study, to those given by the method 3050B given by USEPA [38] and those used by Roca-Perez et al. [39].

| Element | Method of this study | USEPA [38] | Roca-Perez et al. [39] |
| --- | --- | --- | --- |
| Al | 108±6 | na | na |
| As | 79±3 | 102 | 85±6 |
| Cd | 85±4 | 99 | 103±2 |
| Co | 91±6 | 105 | 90±3 |
| Cu | 92±2 | 94 | 90±2 |
| Fe | 99±8 | na | 99±2 |
| Mn | 107±2 | na | 105±3 |
| Mo | 68±2 | 96 | na |
| Pb | 93±9 | 95 | 92±5 |
| Se | 64±2 | 91 | 94±8 |
| Zn | 94±9 | 95 | 91±1 |

All values are percent recovery ±SD; n = 2; na = data not available.
