# Supplementary material for: Soil features in rookeries of Antarctic penguins reveal sea to land biotransport of chemical pollutants
Source: PLoS One. 2017 Aug 16;12(8):e0181901. doi: 10.1371/journal.pone.0181901 (PMC5558944; doi:10.1371/journal.pone.0181901)
Supplement: S3 Table — R stands for each replicate. (DOCX) [file pone.0181901.s003.docx]

**S3 Table.** Raw data for soil variables given in table 2. R stands for each replicate.

| **pH** | **R1** | **R2** | **R3** | **R4** | **R5** | **mean** | **std desv** |
| --- | --- | --- | --- | --- | --- | --- | --- |
| **BY1** | 7.26 | 7.06 |  |  |  | ***7.16*** | ***0.14*** |
| **BY2** | 5.21 | 4.98 |  |  |  | ***5.10*** | ***0.16*** |
| **BY3** | 5.39 | 5.32 |  |  |  | ***5.36*** | ***0.05*** |
| **BY4** | 5.22 | 5.03 |  |  |  | ***5.13*** | ***0.13*** |
| **BY5** | 5.99 | 5.78 |  |  |  | ***5.89*** | ***0.15*** |
| **BY6** | 6.01 | 6.20 | 6.32 |  |  | ***6.18*** | ***0.16*** |
| **PH1** | 5.60 | 5.35 |  |  |  | ***5.48*** | ***0.18*** |
| **PH2** | 6.33 | 6.20 | 6.09 |  |  | ***6.21*** | ***0.12*** |
| **PH3** | 7.28 | 7.54 |  |  |  | ***7.41*** | ***0.18*** |
| **PH4** | 9.43 | 9.23 |  |  |  | ***9.33*** | ***0.14*** |
| **BR1** | 6.85 | 6.63 |  |  |  | ***6.74*** | ***0.16*** |
| **BR2** | 4.96 | 5.07 |  |  |  | ***5.02*** | ***0.08*** |
| **BR3** | 6.54 | 6.78 |  |  |  | ***6.66*** | ***0.17*** |
| **BR4** | 6.90 | 6.72 |  |  |  | ***6.81*** | ***0.13*** |
| **BR5** | 6.35 | 6.21 |  |  |  | ***6.28*** | ***0.10*** |
| **CV1** | 7.45 | 7.67 |  |  |  | ***7.56*** | ***0.16*** |
| **CV2** | 6.98 | 7.18 |  |  |  | ***7.08*** | ***0.14*** |
| **CV3** | 6.44 | 6.24 |  |  |  | ***6.34*** | ***0.14*** |
| **CV4** | 7.12 | 7.32 |  |  |  | ***7.22*** | ***0.14*** |
| **CV5** | 7.31 | 7.06 |  |  |  | ***7.19*** | ***0.18*** |
| **CV6** | 6.05 | 6.13 |  |  |  | ***6.09*** | ***0.06*** |
| **CV7** | 6.23 | 6.01 |  |  |  | ***6.12*** | ***0.16*** |
| **CV8** | 6.45 | 6.30 |  |  |  | ***6.38*** | ***0.11*** |
| **CV9** | 6.46 | 6.08 |  |  |  | ***6.27*** | ***0.27*** |
| **CV10** | 6.33 | 6.62 |  |  |  | ***6.48*** | ***0.21*** |
| **CV11** | 6.46 | 6.06 |  |  |  | ***6.26*** | ***0.28*** |
| **CV12** | 5.89 | 6.10 |  |  |  | ***6.00*** | ***0.15*** |
| **MB1** | 7.55 | 7.45 | 7.40 | 7.31 |  | ***7.43*** | ***0.10*** |
| **MB2** | 7.10 | 7.32 | 7.32 | 7.33 | 7.44 | ***7.30*** | ***0.12*** |
| **MB3** | 6.94 | 6.82 | 6.83 | 6.70 |  | ***6.82*** | ***0.10*** |
| **MB4** | 5.55 | 5.34 |  |  |  | ***5.45*** | ***0.15*** |
| **MB5** | 7.00 | 6.84 | 6.78 | 6.63 |  | ***6.81*** | ***0.15*** |
| **MB6** | 6.64 | 6.81 |  |  |  | ***6.73*** | ***0.12*** |
| **PM1** | 7.41 | 7.64 |  |  |  | ***7.53*** | ***0.16*** |
| **PM2** | 6.70 | 6.51 |  |  |  | ***6.61*** | ***0.13*** |
| **PM3** | 7.79 | 8.03 |  |  |  | ***7.91*** | ***0.17*** |
| **PE1** | 5.58 | 5.59 |  |  |  | ***5.59*** | ***0.01*** |
| **PE2** | 6.09 | 6.22 |  |  |  | ***6.16*** | ***0.09*** |
| **PE3** | 8.18 | 8.00 |  |  |  | ***8.09*** | ***0.13*** |
| **PE4** | 6.12 | 6.02 |  |  |  | ***6.07*** | ***0.07*** |
| **CC1** | 5.23 | 5.02 |  |  |  | ***5.13*** | ***0.15*** |
| **CC2** | 6.69 | 6.59 |  |  |  | ***6.64*** | ***0.07*** |
| **CU1** | 6.64 | 6.63 |  |  |  | ***6.64*** | ***0.01*** |
| **CU2** | 6.46 | 6.21 |  |  |  | ***6.34*** | ***0.18*** |
| **RO1** | 5.65 | 5.45 |  |  |  | ***5.55*** | ***0.14*** |
| **RO2** | 6.44 | 6.65 |  |  |  | ***6.55*** | ***0.15*** |
|  |  |  |  |  |  |  |  |
| **EC** | **R1** | **R2** | **R3** | **R4** | **mean** | **std desv** |  |
| **BY1** | 2.561 | 2.460 |  |  | ***2.51*** | ***0.07*** |  |
| **BY2** | 0.211 | 0.190 |  |  | ***0.20*** | ***0.01*** |  |
| **BY3** | 0.409 | 0.390 |  |  | ***0.40*** | ***0.01*** |  |
| **BY4** | 0.261 | 0.250 |  |  | ***0.26*** | ***0.01*** |  |
| **BY5** | 0.020 | 0.030 |  |  | ***0.02*** | ***0.01*** |  |
| **BY6** | 1.197 | 1.100 |  |  | ***1.15*** | ***0.07*** |  |
| **PH1** | 3.563 | 3.401 |  |  | ***3.48*** | ***0.11*** |  |
| **PH2** | 0.986 | 0.957 |  |  | ***0.97*** | ***0.02*** |  |
| **PH3** | 0.260 | 0.280 |  |  | ***0.27*** | ***0.01*** |  |
| **PH4** | 0.150 | 0.170 |  |  | ***0.16*** | ***0.01*** |  |
| **BR1** | 0.153 | 0.140 |  |  | ***0.15*** | ***0.01*** |  |
| **BR2** | 0.341 | 0.310 |  |  | ***0.33*** | ***0.02*** |  |
| **BR3** | 4.571 | 3.972 |  |  | ***4.27*** | ***0.42*** |  |
| **BR4** | 0.188 | 0.180 |  |  | ***0.18*** | ***0.01*** |  |
| **BR5** | 3.690 | 3.220 |  |  | ***3.46*** | ***0.33*** |  |
| **CV1** | 0.927 | 0.941 |  |  | ***0.93*** | ***0.01*** |  |
| **CV2** | 3.610 | 3.500 |  |  | ***3.56*** | ***0.08*** |  |
| **CV3** | 6.330 | 6.200 |  |  | ***6.27*** | ***0.09*** |  |
| **CV4** | 3.358 | 3.410 |  |  | ***3.38*** | ***0.04*** |  |
| **CV5** | 0.227 | 0.228 | 0.210 |  | ***0.22*** | ***0.01*** |  |
| **CV6** | 0.281 | 0.282 | 0.270 |  | ***0.28*** | ***0.01*** |  |
| **CV7** | 0.811 | 0.700 | 0.810 |  | ***0.77*** | ***0.06*** |  |
| **CV8** | 0.220 | 0.210 |  |  | ***0.22*** | ***0.01*** |  |
| **CV9** | 0.180 | 0.190 |  |  | ***0.19*** | ***0.01*** |  |
| **CV10** | 0.082 | 0.070 |  |  | ***0.08*** | ***0.01*** |  |
| **CV11** | 0.115 | 0.115 |  |  | ***0.12*** | ***0.00*** |  |
| **CV12** | 0.209 | 0.200 |  |  | ***0.20*** | ***0.01*** |  |
| **MB1** | 0.720 | 0.823 | 0.818 | 0.920 | ***0.82*** | ***0.08*** |  |
| **MB2** | 0.630 | 0.589 | 0.534 | 0.510 | ***0.57*** | ***0.05*** |  |
| **MB3** | 2.650 | 2.756 | 3.000 | 2.907 | ***2.83*** | ***0.16*** |  |
| **MB4** | 0.254 | 0.240 |  |  | ***0.25*** | ***0.01*** |  |
| **MB5** | 2.940 | 3.050 | 2.740 | 3.069 | ***2.95*** | ***0.15*** |  |
| **MB6** | 0.147 | 0.140 |  |  | ***0.14*** | ***0.00*** |  |
| **PM1** | 0.414 | 0.400 |  |  | ***0.41*** | ***0.01*** |  |
| **PM2** | 2.274 | 2.200 |  |  | ***2.24*** | ***0.05*** |  |
| **PM3** | 0.294 | 0.280 |  |  | ***0.29*** | ***0.01*** |  |
| **PE1** | 0.275 | 0.276 | 0.260 |  | ***0.27*** | ***0.01*** |  |
| **PE2** | 8.837 | 7.837 |  |  | ***8.34*** | ***0.71*** |  |
| **PE3** | 0.231 | 0.200 | 0.190 | 0.220 | ***0.21*** | ***0.02*** |  |
| **PE4** | 8.220 | 8.380 |  |  | ***8.30*** | ***0.11*** |  |
| **CC1** | 0.492 | 0.430 |  |  | ***0.46*** | ***0.04*** |  |
| **CC2** | 1.474 | 1.379 |  |  | ***1.43*** | ***0.07*** |  |
| **CU1** | 3.206 | 3.011 |  |  | ***3.11*** | ***0.14*** |  |
| **CU2** | 0.101 | 0.115 |  |  | ***0.11*** | ***0.01*** |  |
| **RO1** | 0.360 | 0.341 |  |  | ***0.35*** | ***0.01*** |  |
| **RO2** | 3.945 | 3.752 |  |  | ***3.85*** | ***0.14*** |  |
|  |  |  |  |  |  |  |  |
| **%Corg** | **R1** | **R2** | **mean** | **std desv** |  |  |  |
| **BY1** | 5.97 | 5.99 | ***5.98*** | ***0.01*** |  |  |  |
| **BY2** | 3.10 | 3.20 | ***3.15*** | ***0.07*** |  |  |  |
| **BY3** | 6.50 | 6.46 | ***6.48*** | ***0.03*** |  |  |  |
| **BY4** | 0.81 | 0.83 | ***0.82*** | ***0.01*** |  |  |  |
| **BY5** | 19.30 | 18.80 | ***19.05*** | ***0.35*** |  |  |  |
| **BY6** | 3.95 | 4.05 | ***4.00*** | ***0.07*** |  |  |  |
| **PH1** | 20.50 | 20.30 | ***20.40*** | ***0.14*** |  |  |  |
| **PH2** | 18.40 | 18.30 | ***18.35*** | ***0.07*** |  |  |  |
| **PH3** | 0.68 | 0.70 | ***0.69*** | ***0.01*** |  |  |  |
| **PH4** | 0.13 | 0.13 | ***0.13*** | ***0.00*** |  |  |  |
| **BR1** | 1.23 | 1.29 | ***1.26*** | ***0.04*** |  |  |  |
| **BR2** | 2.90 | 2.98 | ***2.94*** | ***0.06*** |  |  |  |
| **BR3** | 7.39 | 7.43 | ***7.41*** | ***0.03*** |  |  |  |
| **BR4** | 1.05 | 1.10 | ***1.08*** | ***0.04*** |  |  |  |
| **BR5** | 7.80 | 8.18 | ***7.99*** | ***0.27*** |  |  |  |
| **CV1** | 0.69 | 0.71 | ***0.70*** | ***0.01*** |  |  |  |
| **CV2** | 5.71 | 5.76 | ***5.74*** | ***0.04*** |  |  |  |
| **CV3** | 10.19 | 10.70 | ***10.45*** | ***0.36*** |  |  |  |
| **CV4** | 1.49 | 1.53 | ***1.51*** | ***0.03*** |  |  |  |
| **CV5** | 0.23 | 0.24 | ***0.23*** | ***0.01*** |  |  |  |
| **CV6** | 0.93 | 0.89 | ***0.91*** | ***0.02*** |  |  |  |
| **CV7** | 2.12 | 2.19 | ***2.16*** | ***0.05*** |  |  |  |
| **CV8** | 0.54 | 0.56 | ***0.55*** | ***0.01*** |  |  |  |
| **CV9** | 0.42 | 0.44 | ***0.43*** | ***0.01*** |  |  |  |
| **CV10** | 0.56 | 0.58 | ***0.57*** | ***0.01*** |  |  |  |
| **CV11** | 0.34 | 0.35 | ***0.34*** | ***0.01*** |  |  |  |
| **CV12** | 0.45 | 0.45 | ***0.45*** | ***0.00*** |  |  |  |
| **MB1** | 0.66 | 0.68 | ***0.67*** | ***0.01*** |  |  |  |
| **MB2** | 0.66 | 0.67 | ***0.66*** | ***0.01*** |  |  |  |
| **MB3** | 2.40 | 2.46 | ***2.43*** | ***0.04*** |  |  |  |
| **MB4** | 0.97 | 0.95 | ***0.96*** | ***0.01*** |  |  |  |
| **MB5** | 2.60 | 2.74 | ***2.67*** | ***0.10*** |  |  |  |
| **MB6** | 0.22 | 0.21 | ***0.21*** | ***0.00*** |  |  |  |
| **PM1** | 0.64 | 0.66 | ***0.65*** | ***0.01*** |  |  |  |
| **PM2** | 1.70 | 1.78 | ***1.74*** | ***0.06*** |  |  |  |
| **PM3** | 0.28 | 0.29 | ***0.28*** | ***0.00*** |  |  |  |
| **PE1** | 0.50 | 0.52 | ***0.51*** | ***0.01*** |  |  |  |
| **PE2** | 7.20 | 7.22 | ***7.21*** | ***0.01*** |  |  |  |
| **PE3** | 0.32 | 0.33 | ***0.32*** | ***0.01*** |  |  |  |
| **PE4** | 6.90 | 6.96 | ***6.93*** | ***0.04*** |  |  |  |
| **CC1** | 23.80 | 24.00 | ***23.90*** | ***0.14*** |  |  |  |
| **CC2** | 23.50 | 23.30 | ***23.40*** | ***0.14*** |  |  |  |
| **CU1** | 11.00 | 10.50 | ***10.75*** | ***0.35*** |  |  |  |
| **CU2** | 8.30 | 8.20 | ***8.25*** | ***0.07*** |  |  |  |
| **RO1** | 12.20 | 12.40 | ***12.30*** | ***0.14*** |  |  |  |
| **RO2** | 9.45 | 9.55 | ***9.50*** | ***0.07*** |  |  |  |
|  |  |  |  |  |  |  |  |
| **%C** | **R1** | **R2** | **mean** | **std desv** |  |  |  |
| **BY1** | 6.27 | 6.47 | ***6.37*** | ***0.14*** |  |  |  |
| **BY2** | 3.22 | 3.08 | ***3.15*** | ***0.10*** |  |  |  |
| **BY3** | 7.50 | 7.20 | ***7.35*** | ***0.21*** |  |  |  |
| **BY4** | 0.80 | 0.84 | ***0.82*** | ***0.03*** |  |  |  |
| **BY5** | 20.45 | 19.64 | ***20.04*** | ***0.57*** |  |  |  |
| **BY6** | 4.32 | 4.52 | ***4.42*** | ***0.14*** |  |  |  |
| **PH1** | 21.04 | 20.79 | ***20.91*** | ***0.18*** |  |  |  |
| **PH2** | 18.64 | 19.41 | ***19.03*** | ***0.54*** |  |  |  |
| **PH3** | 0.72 | 0.76 | ***0.74*** | ***0.03*** |  |  |  |
| **PH4** | 0.13 | 0.13 | ***0.13*** | ***0.00*** |  |  |  |
| **BR1** | 1.24 | 1.30 | ***1.27*** | ***0.04*** |  |  |  |
| **BR2** | 3.20 | 3.40 | ***3.30*** | ***0.14*** |  |  |  |
| **BR3** | 7.32 | 7.52 | ***7.42*** | ***0.14*** |  |  |  |
| **BR4** | 1.10 | 1.16 | ***1.13*** | ***0.04*** |  |  |  |
| **BR5** | 7.92 | 8.45 | ***8.19*** | ***0.37*** |  |  |  |
| **CV1** | 0.71 | 0.75 | ***0.73*** | ***0.03*** |  |  |  |
| **CV2** | 5.88 | 6.08 | ***5.98*** | ***0.14*** |  |  |  |
| **CV3** | 10.20 | 10.70 | ***10.45*** | ***0.35*** |  |  |  |
| **CV4** | 1.49 | 1.59 | ***1.54*** | ***0.07*** |  |  |  |
| **CV5** | 0.23 | 0.24 | ***0.23*** | ***0.01*** |  |  |  |
| **CV6** | 0.93 | 0.89 | ***0.91*** | ***0.03*** |  |  |  |
| **CV7** | 2.16 | 2.26 | ***2.21*** | ***0.07*** |  |  |  |
| **CV8** | 0.54 | 0.56 | ***0.55*** | ***0.01*** |  |  |  |
| **CV9** | 0.44 | 0.46 | ***0.45*** | ***0.01*** |  |  |  |
| **CV10** | 0.56 | 0.58 | ***0.57*** | ***0.01*** |  |  |  |
| **CV11** | 0.34 | 0.36 | ***0.35*** | ***0.01*** |  |  |  |
| **CV12** | 0.46 | 0.48 | ***0.47*** | ***0.01*** |  |  |  |
| **MB1** | 0.73 | 0.77 | ***0.75*** | ***0.03*** |  |  |  |
| **MB2** | 0.66 | 0.67 | ***0.66*** | ***0.01*** |  |  |  |
| **MB3** | 2.77 | 2.97 | ***2.87*** | ***0.14*** |  |  |  |
| **MB4** | 0.97 | 0.99 | ***0.98*** | ***0.01*** |  |  |  |
| **MB5** | 2.69 | 2.88 | ***2.78*** | ***0.14*** |  |  |  |
| **MB6** | 0.24 | 0.23 | ***0.23*** | ***0.01*** |  |  |  |
| **PM1** | 0.64 | 0.66 | ***0.65*** | ***0.01*** |  |  |  |
| **PM2** | 1.79 | 1.81 | ***1.80*** | ***0.01*** |  |  |  |
| **PM3** | 0.28 | 0.29 | ***0.28*** | ***0.01*** |  |  |  |
| **PE1** | 0.50 | 0.53 | ***0.51*** | ***0.02*** |  |  |  |
| **PE2** | 7.11 | 7.32 | ***7.21*** | ***0.14*** |  |  |  |
| **PE3** | 0.38 | 0.40 | ***0.39*** | ***0.01*** |  |  |  |
| **PE4** | 7.00 | 7.18 | ***7.09*** | ***0.13*** |  |  |  |
| **CC1** | 24.19 | 24.04 | ***24.12*** | ***0.11*** |  |  |  |
| **CC2** | 24.04 | 24.00 | ***24.02*** | ***0.02*** |  |  |  |
| **CU1** | 11.30 | 10.61 | ***10.96*** | ***0.49*** |  |  |  |
| **CU2** | 8.70 | 8.90 | ***8.80*** | ***0.14*** |  |  |  |
| **RO1** | 12.10 | 12.50 | ***12.30*** | ***0.28*** |  |  |  |
| **RO2** | 9.50 | 9.50 | ***9.50*** | ***0.00*** |  |  |  |
|  |  |  |  |  |  |  |  |
| **%N** | **R1** | **R2** | **mean** | **std desv** |  |  |  |
| **BY1** | 2.49 | 2.55 | ***2.52*** | ***0.04*** |  |  |  |
| **BY2** | 0.53 | 0.50 | ***0.52*** | ***0.02*** |  |  |  |
| **BY3** | 1.30 | 1.33 | ***1.32*** | ***0.02*** |  |  |  |
| **BY4** | 0.30 | 0.28 | ***0.29*** | ***0.02*** |  |  |  |
| **BY5** | 8.48 | 8.36 | ***8.42*** | ***0.09*** |  |  |  |
| **BY6** | 1.18 | 1.20 | ***1.19*** | ***0.02*** |  |  |  |
| **PH1** | 10.15 | 9.88 | ***10.01*** | ***0.19*** |  |  |  |
| **PH2** | 10.42 | 10.37 | ***10.40*** | ***0.04*** |  |  |  |
| **PH3** | 0.29 | 0.25 | ***0.27*** | ***0.03*** |  |  |  |
| **PH4** | 0.10 | 0.11 | ***0.10*** | ***0.01*** |  |  |  |
| **BR1** | 0.28 | 0.24 | ***0.26*** | ***0.03*** |  |  |  |
| **BR2** | 0.58 | 0.51 | ***0.54*** | ***0.05*** |  |  |  |
| **BR3** | 3.34 | 3.41 | ***3.38*** | ***0.05*** |  |  |  |
| **BR4** | 0.29 | 0.31 | ***0.30*** | ***0.01*** |  |  |  |
| **BR5** | 3.21 | 2.99 | ***3.10*** | ***0.15*** |  |  |  |
| **CV1** | 0.34 | 0.30 | ***0.32*** | ***0.03*** |  |  |  |
| **CV2** | 2.56 | 2.51 | ***2.53*** | ***0.04*** |  |  |  |
| **CV3** | 5.28 | 5.16 | ***5.22*** | ***0.09*** |  |  |  |
| **CV4** | 0.63 | 0.66 | ***0.64*** | ***0.02*** |  |  |  |
| **CV5** | 0.12 | 0.10 | ***0.11*** | ***0.02*** |  |  |  |
| **CV6** | 0.21 | 0.25 | ***0.23*** | ***0.02*** |  |  |  |
| **CV7** | 0.53 | 0.50 | ***0.52*** | ***0.02*** |  |  |  |
| **CV8** | 0.18 | 0.20 | ***0.19*** | ***0.01*** |  |  |  |
| **CV9** | 0.11 | 0.12 | ***0.11*** | ***0.01*** |  |  |  |
| **CV10** | 0.15 | 0.17 | ***0.16*** | ***0.01*** |  |  |  |
| **CV11** | 0.13 | 0.11 | ***0.12*** | ***0.01*** |  |  |  |
| **CV12** | 0.17 | 0.19 | ***0.18*** | ***0.02*** |  |  |  |
| **MB1** | 0.28 | 0.25 | ***0.27*** | ***0.02*** |  |  |  |
| **MB2** | 0.24 | 0.21 | ***0.23*** | ***0.02*** |  |  |  |
| **MB3** | 0.80 | 0.75 | ***0.78*** | ***0.04*** |  |  |  |
| **MB4** | 0.24 | 0.25 | ***0.25*** | ***0.01*** |  |  |  |
| **MB5** | 0.83 | 0.77 | ***0.80*** | ***0.04*** |  |  |  |
| **MB6** | 0.10 | 0.11 | ***0.10*** | ***0.01*** |  |  |  |
| **PM1** | 0.22 | 0.20 | ***0.21*** | ***0.02*** |  |  |  |
| **PM2** | 0.90 | 0.92 | ***0.91*** | ***0.02*** |  |  |  |
| **PM3** | 0.14 | 0.12 | ***0.13*** | ***0.02*** |  |  |  |
| **PE1** | 0.22 | 0.20 | ***0.21*** | ***0.01*** |  |  |  |
| **PE2** | 3.49 | 3.54 | ***3.51*** | ***0.04*** |  |  |  |
| **PE3** | 0.08 | 0.06 | ***0.07*** | ***0.02*** |  |  |  |
| **PE4** | 3.01 | 3.09 | ***3.05*** | ***0.05*** |  |  |  |
| **CC1** | 3.18 | 3.23 | ***3.21*** | ***0.03*** |  |  |  |
| **CC2** | 5.04 | 4.91 | ***4.98*** | ***0.09*** |  |  |  |
| **CU1** | 3.79 | 3.80 | ***3.79*** | ***0.01*** |  |  |  |
| **CU2** | 1.30 | 1.33 | ***1.32*** | ***0.02*** |  |  |  |
| **RO1** | 1.32 | 1.29 | ***1.31*** | ***0.02*** |  |  |  |
| **RO2** | 3.81 | 3.72 | ***3.76*** | ***0.06*** |  |  |  |
